# True cyst formation underlies persistence and drug tolerance in *Tritrichomonas foetus*

**DOI:** 10.1038/s41467-026-71827-9

**Published:** 2026-04-28

**Authors:** Lucrecia S. Iriarte, Andres M. Alonso, Natalia M. Villarreal, Cristian I. Martinez, Maria S. Prandi, Luiz D. Barros, Marcos Ferrante, Natalia de Miguel, Veronica M. Coceres

**Affiliations:** 1 Laboratorio de Parásitos Anaerobios, Instituto Tecnológico Chascomús (INTECH), CONICET-UNSAM, Chascomús, Argentina; 2 EByN, Escuela de Bio y Nanotecnologías (UNSAM), San Martín, Buenos Aires, Argentina; 3 Laboratorio de Bioquímica y Fisiología de la Maduración de Frutos, Instituto Tecnológico Chascomús (INTECH), CONICET-UNSAM, Chascomús, Argentina; 4 Department of Veterinary Medicine, Universidade Federal de Lavras-UFLA, Lavras, MG Brazil

**Keywords:** Parasite biology, Mechanisms of disease, Parasite development

## Abstract

*Tritrichomonas foetus*, a protozoan parasite responsible for bovine and feline trichomonosis, has traditionally been considered to form only pseudocysts. Here, we demonstrate that *T. foetus* produces true cysts characterized by a chitin-like, fibrillar wall, cytoskeletal reorganization, and resistance to detergent treatment. Using scanning and transmission electron microscopy, sarkosyl resistance assays, lectin and chitin-binding staining, and monosaccharide composition analysis, we identify a polysaccharide-rich cyst wall enriched in N-acetylated hexosamines. Cystogenesis is induced by environmental stresses, including nutrient deprivation, alkaline pH mimicking the bovine preputial environment, and metronidazole exposure. Flow cytometry analyses reveal increased DNA content and multinucleation in cysts, consistent with endoreplication. Encysted parasites remain viable and revert to proliferative trophozoites upon restoration of favorable conditions. Pharmacological inhibition of cyst wall biosynthesis enhances metronidazole susceptibility, supporting a functional role for cyst formation in drug tolerance. Transcriptomic profiling identifies pathways associated with carbohydrate metabolism, Myb-like transcription factors, and calcium/calmodulin-dependent kinases during encystation. Together, our findings establish true cyst formation in *T. foetus* and define a previously unrecognized survival state linked to environmental persistence and reduced drug susceptibility.

## Abstract (web summary)

In this study, Veronica Coceres and colleagues show that under stress, parasite *Tritrichomonas foetus* forms true cysts that reduce drug susceptibility. This study reveals a previously unknown parasite survival strategy with implications for persistence and transmission.

## Introduction

Trichomonads are anaerobic flagellated protists that are either parasites or commensals, generally living in the genitourinary or digestive tract of animals and humans^1^. Among the trichomonads, *Tritrichomonas foetus* is considered the species of major veterinary importance, causing reproductive problems in cattle, leading to substantial economic losses due to reproductive failures^2^. The life cycle of *T. foetus* is known as monoxenous; thus, development is restricted to a single host species. In bovines, trichomonosis has traditionally been considered as a venereal disease. However, recent findings indicate that the protozoan can survive the passage through the bovine gastrointestinal tract. In animals infected orally, parasites can be discharged in feces and contaminate the cow’s reproductive tract, facilitated by the ventral anatomical position of the vagina^3^. Similarly, *T. foetus* and *Pentatrichomonas hominis,* a human pathogen, have recently been reported as causes  of chronic diarrhea in domestic animals^4,5^ and have even been isolated from samples obtained from wild animals^6^. Highlighting the adaptability of these protists  to diverse environments, Martinez et al. demonstrated that *T. foetus* can survive for several days in bovine feces and water, probably through the formation of resistant pseudocysts or cyst-like structures^3^.

It is now widely accepted that *T. foetus* has two cellular forms: flagellated trophozoites and pseudocysts. Pseudocysts lack a true cyst wall and are formed when trophozoites internalize their flagella in response to unfavorable environmental conditions^7^. Although their precise role in the life cycle of Tritrichomonadidae remains unclear, pseudocysts are generally considered part of the protozoan’s environmental stress response. Similar stress-induced forms have been described in other trichomonads residing in the gastrointestinal tract^8,9^. While the infective capacity of pseudocysts in many trichomonads remains unclear, fecal pseudocysts have been shown to be infective in rodents and birds^10–12^. In contrast, true cysts (with a defined cyst wall) have been identified in several intestinal trichomonads, including *Trichomitus batrachorum*, *Trichomitus sanguisugae* and in the free-living trichomonad *Ditrichomonas honigbergii*^13^. Moreover, cyst formation has been observed in other members of the phylum Parabasalia, including monocercomonads and hypermastigotes^14^. Similar survival adaptations have been reported in other protozoa that form cysts during their life cycle. For instance, several free-living marine amoebae form pseudocysts as a common short-term survival strategy to escape from undesirable salinity variations.

In this study, we report the formation of true cysts by *T. foetus* in response to nutritional stress and alkaline pH conditions. Moreover, we provide evidence that cyst formation may contribute to *T. foetus* resistance to metronidazole treatment. Taken together, our results demonstrate that *T. foetus* can form true cysts under certain conditions, which could be relevant for the life cycle of this parasite in bovines and future alternative therapeutic strategies design.

## Results

### Nutritional stress induces true cysts in *T. foetus*

**Fig. 1:**
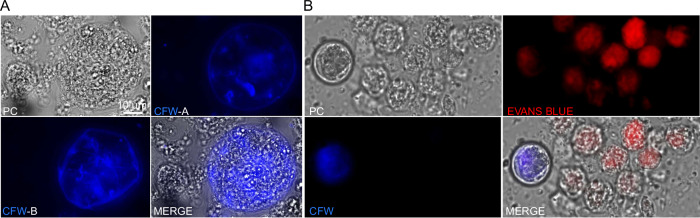
Analysis of *T. foetus*cyst-like structures. **A** Representative image of a *T. foetus* cyst-like structure incubated under nutrient starvation conditions and stained with Calcofluor white (CFW). Samples were analyzed by epifluorescence microscopy. PC, phase-contrast image. CFW A-B, different focal planes of the same structure. Scale bar: 10 µm. **B** Representative image of cyst-like structures incubated under nutrient starvation conditions and stained with a premixed solution of Calcofluor White (CFW, blue) and Evans Blue (red). Parasites were visualized using fluorescence microscope. PC, phase-contrast image. Scale bar: 10 µm. Images are representative of three independent biological experiments.

The formation of cyst-like structures in *T. foetus* under stress conditions has been proposed in recent studies^3^. Considering that pseudocysts may represent an intermediate stage in cyst maturation, a process influenced by the duration of stress, we evaluated the formation of mature cyst-like structures in *T. foetus* exposed to prolonged nutritional deprivation^15,16^. To this end, trophozoites were incubated in TYM culture media lacking maltose and horse serum supplementation for 96 hours and the presence of pseudocysts or cyst-like structures was evaluated by Calcofluor White (CFW) staining. CFW is commonly used to stain and detect cysts of certain parasites, particularly those that have chitin or cellulose in their cyst walls^17–19^. As observed in Fig. 1A, we detected CFW-positive structures by fluorescence microscopy, suggesting the presence of chitin- or cellulose-containing components in the membrane of *T. foetus* incubated under nutritional stress, indicative of cyst-like structures. To demonstrate that CFW-positive structures are cell membrane impermeable, we employed a dual-staining approach using premixed Calcofluor white (CFW) and Evans blue (EB) dyes. Using this combined staining technique, we detected both EB + /CFW− and CFW + /EB− structures in parasites maintained under nutritional stress conditions for 48 hours (Fig. 1B). These findings suggest the formation  of a true cyst wall in a subset of parasites exposed to these stress conditions.

**Fig. 2:**
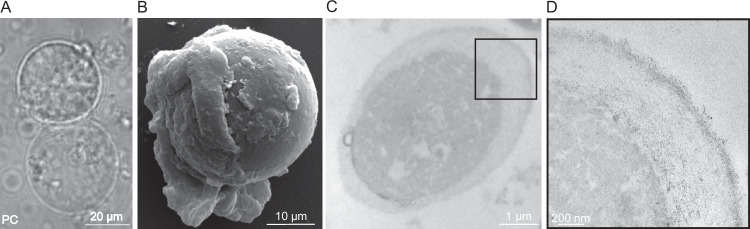
*T. foetus* forms true cysts. **A** Image of *T. foetus* cyst-like structures resistant to sarkosyl detergent. PC, phase-contrast image. Scale bar: 20 µm. **B** Scanning electron microscopy (SEM) image showing the external morphology of a cyst. Scale bar: 5 µm. **C** Transmission electron microscopy (TEM) image of a cyst revealing internal organization and a distinct multilayered cyst wall. Scale bar: 1 µm. **D** Magnified TEM view of the boxed area, highlighting the layered architecture of the cyst wall. Scale bar: 200 nm. Images are representative of three independent biological experiments.

Considering that cyst forms are typically resistant to ionic detergents, *T. foetus* parasites incubated under nutritional stress conditions for 48 hours were treated with 0.15% sarkosyl to eliminate the trophozoites and/or motile forms^20^. Then, the presence of sarkosyl-resistant structures was analyzed by light microscopy (Fig. 2A) and electron microscopy (Fig. 2B, C). Scanning electron microscopy revealed the surface features of cyst-like structures (Fig. 2B), while transmission electron microscopy confirmed the presence of a fibrillar cyst wall measuring approximately 0.337 ± 0.07 µm in thickness (Fig. 2C, D). Together, these results demonstrate that *T. foetus* forms cyst structures with true walls in response to nutritional stress.

Additionally, we characterized the population dynamics during the encystation process using CFW and Evans blue staining in parasites maintained under nutritional stress. Early-stage parasites showed strong EB uptake together with diffuse CFW labeling (CFW⁺/EB⁺ population). Intermediate forms exhibited partial EB exclusion and a more defined CFW-positive wall, whereas fully developed structures completely excluded EB and displayed intense CFW fluorescence (CFW⁺/EB⁻ population). CFW⁺/EB⁻ structures were resistant to sarkosyl; therefore, we classified them as mature cysts^19^. (Supplementary Fig. 1).

**Fig. 3:**
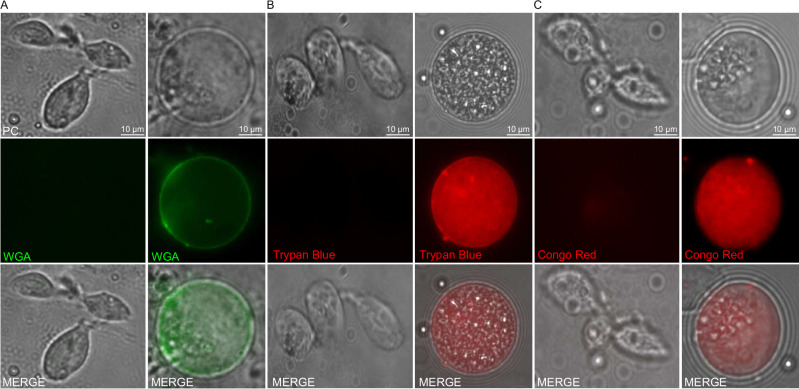
Surface characterization of *T. foetus* cysts using fluorescence staining. Surface glycoconjugates in trophozoites and cysts were analyzed using wheat germ agglutinin (WGA) **A**, Trypan Blue **B** and Congo Red **C**. PC, phase-contrast image. Scale bars: 10 µm. Images are representative of three independent biological experiments.

In some protozoan parasites, such as Entamoeba and Giardia, the cyst wall (CW) is 120-150 nm thick and is composed of polysaccharides associated with a variable number of proteins, some of which have lectin properties^21^. Chitin-binding dyes, including Calcofluor White, Wheat Germ Agglutinin (WGA), Congo Red, and Trypan Blue, are commonly used to label cyst wall in fungi and cyst-forming protozoa. Specifically, CFW binds nonspecifically to beta-1,3 and 1,4-linked polysaccharides, including chitin^19,22^. WGA specifically binds to N-acetyl-D-glucosamine, i.e., the monomeric unit of chitin. Congo Red is known to bind chitin and β (1,3)-glucan fibers^23,24^ while Trypan Blue emits strong red fluorescence upon binding to chitin and yeast glucan^25^. In this context, we employed several chitin-binding dyes (WGA, Trypan Blue, and Congo Red) to analyze the presence of chitin-like polysaccharides in trophozoites and sarkosyl-resistant cysts. A strong signal for WGA, Trypan Blue, and Congo Red was observed in sarkosyl-resistant cysts, whereas such staining was not detected in trophozoites or motile forms (Fig. 3A–C). These results suggested the presence of specific polysaccharides in cysts of *T. foetus*, similar to those found in the cyst wall of other protozoan parasites.

**Fig. 4:**
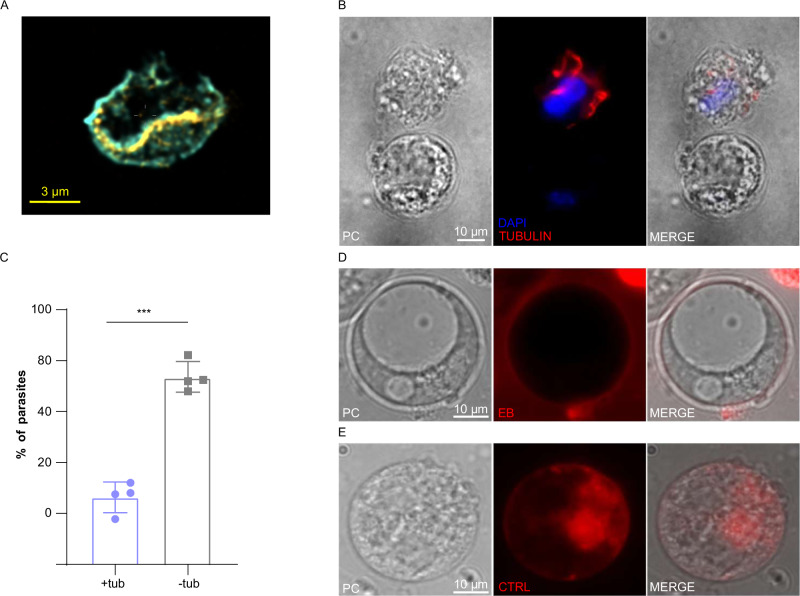
Internal structural analysis of *T. foetus* cysts. **A** Confocal image of indirect immunofluorescence using an anti-alpha tubulin antibody showing spherical parasites with internalized flagella (yellow). Scale bar: 3 µm. **B** Indirect immunofluorescence image showing alpha tubulin (red) of *T. foetus* cysts. Nuclei were counterstained with DAPI (blue). PC, phase-contrast image. Images are representative of three independent biological experiments. **C** Quantitative analysis of tubulin staining in cysts. PC, phase-contrast image. Bars represent mean ± s.d. from three independent experiments (n = 3 biological replicates). All individual data points are shown. Statistical significance was determined using a two-tailed Student’s t-test (P ≤ 0.0001). Source data are provided as a Source Data file. **D** Representative image of a cyst stained with Evans blue (EB) and Cell Tracker Red (CTR) **E** to assess cytoplasmic content distribution. PC, phase-contrast image. Scale bar: 10 µm. Images are representative of three independent biological experiments.

The encystation process involves dramatic structural reorganization, including ciliature resorption in some species^26^. In *T. foetus*, previous reports indicated  that pseudocyst formation involves loss of cytoskeletal structures^12^. We evaluated the cytoskeleton organization in cysts using an anti-alpha tubulin antibody by fluorescence microscopy. We observed cysts with internalized flagella and curved axostyles^3^, and confirmed this observation by confocal laser scanning microscopy (Fig. 4A). Notably, α-tubulin staining was absent in a subset of cysts, while nuclei were consistently labeled in all observed protozoa during the colocalization assays (Fig. 4B). Quantitative analysis showed that only 26 % of cysts were positive for tubulin staining, and 74% were negative during the colocalization assays (Fig. 4C), suggesting progressive cytoskeleton disassembly during the maturation of the cyst. Additionally, we observed redistribution of cytoplasmatic content in cysts by fluorescence microscopy. This was observed using Evans Blue staining (Fig. 4D) as well as with Cell Tracker Red (Fig. 4E) during the encystation process. These findings may reflect cytoplasmic condensation and a reduction in the cell volume during cyst maturation evolution.

### Alkaline pH induces in vitro encystment of *T. foetus*

**Fig. 5:**
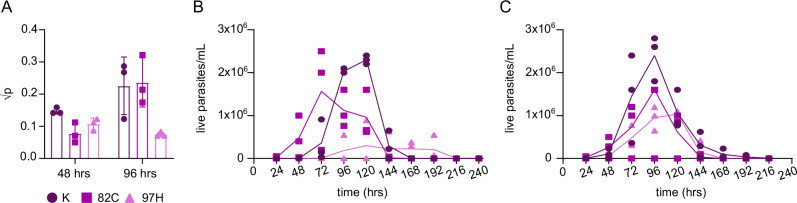
Optimization of *T. foetus* cyst induction in vitro. **A** Quantification of sarkosyl-resistant parasites after incubation under nutrient starvation for 48 and 96 hours in different *T. foetus* strains (K, 82 C, and 97H). Data represent the percentage of sarkosyl-resistant parasites. Proportions were arcsine square-root transformed prior to statistical analysis. Data are presented as mean ± s.d. of the transformed values (n = 3 biological replicates). Percentages are reported in the text for clarity and biological interpretation. Kinetic growth analysis of excysted *T. foetus* parasites in TYM medium following encystment induction under nutrient starvation for 48 hours **B** and 96 hours **C**. All individual data points are shown; the line represents the mean of three independent biological replicates, quantified every 24 hours. Source data are provided as a Source Data file.

To determine whether cyst formation in *T. foetus* is strain-independent, we evaluated the formation of cyst-like structures under diverse stress conditions in three different *T. foetus* strains: K, 82 C, and 97H. Parasites were incubated under nutrient restriction conditions (TYM culture medium without maltose and serum supplementation) for 48 and 96 hours, and the number of sarkosyl-resistant parasites was quantified by light microscopy. Encystment efficiency was calculated as the ratio of sarkosyl-resistant forms to the total number of *T. foetus* initially subjected to the induction process. At 48 hours, the encystment rate were 2,16% for the K strain, 0.66% for 82 C and 1.166% for 97H. At 96 hours, the rates increased to 5.5% for K and 5.83% for C, while 97H strain remained low at 0.566% (Fig. 5A). These results demonstrate that multiple *T. foetus* strains are capable of forming cyst-like structures under nutrient-deprivation, although with varying efficiencies. Since encystment is a reversible process in many protozoa, requiring excystment to regain a proliferative state^27^, we next evaluated the excystment and growth recovery in  the three strains. Induced cysts obtained  at 48 and 96 hours were transferred to standard TYM culture conditions. All strains were able to excyst and replicate, although the 97H strain was less efficient at both time points (Fig. 5B, C). Together, these results confirm that different *T. foetus* strains are  capable of encysting in nutrient restriction conditions and excyst when standard  nutritional conditions are  restored.

**Fig. 6:**
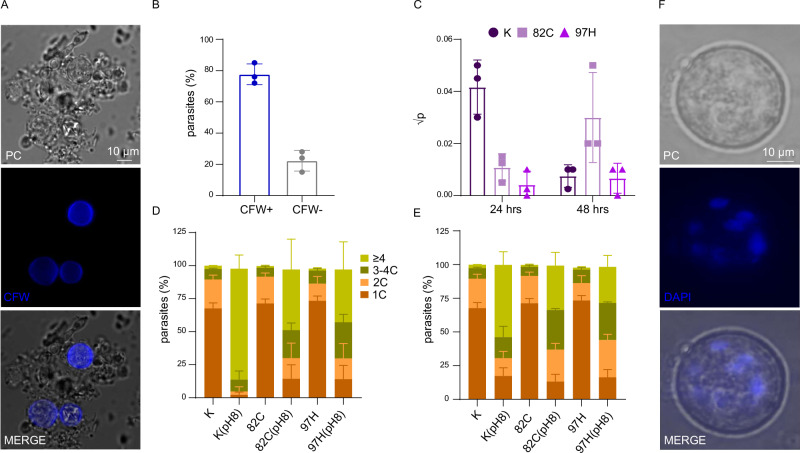
*T. foetus* cysts are induced by alkaline pH conditions. **A** Representative image of *T. foetus* parasites incubated for 48 hours in a preputial mucus sample. Structures were stained with Calcofluor white (blue). PC, phase-contrast image. **B** Quantification of CFW-positive and CFW-negative structures. Bars represent the mean percentage ± s.d. Each dot represents an independent biological replicate. Statistical significance was assessed using a two-tailed unpaired t-test. **C** Quantification of sarkosyl-resistant parasites after incubation in TYM at pH 8 for 24 and 48 hours in different *T. foetus* strains (K, 82 C, and 97H). Data represent the percentage of sarkosyl-resistant parasites. Proportions were arcsine square-root transformed prior to statistical analysis. Data are presented as mean ± s.d. of the transformed values (n = 3 biological replicates). Percentages are shown in the main text for clarity and biological interpretation. **D**, **E** DNA content profiles of *T. foetus* strains (K, 82 C, and 97H) grown in standard culture medium or in TYM at pH 8 for 24 hours **D** and 48 hours **E**, measured by flow cytometry. Results are presented as mean ± s.e.m. of three independent biological experiments. Mean values for each dataset are provided in Table 1. **F** Representative image of multinucleate *T. foetus* K grown in TYM at pH 8 for 48 hours, stained with DAPI (blue) and visualized by epifluorescence microscopy. PC, phase-contrast image. Scale bar, 10 μm. Images are representative of three independent biological experiments. Source data are provided as a Source Data file.

Previous reports demonstrated that approximately 55% of the *T. foetus* present in fresh preputial mucus samples of bulls naturally infected were pseudocystic forms^28^. To evaluate whether some of these structures could be true cysts, we incubated trophozoites in fresh preputial mucus samples for 48 hours, and the presence of cyst was evaluated by CFW staining (Fig. 6A). Our results indicate that after incubation in fresh preputial mucus ~80% of parasites were CFW+ (Fig. 6B), suggesting the presence of cyst-like structures. Considering that one of the most relevant characteristic of bull preputial mucus is its alkalinity (~pH 8.3)^29^, we investigated whether alkalinity could serve as a cyst-inducing stimulus. *T. foetus* strains (K, 82 C, and 97H) were incubated in TYM medium adjusted to pH 8 for 24 and 48 hours. Sarkosyl resistance assays showed that the K strain formed 4.17% and 0.75% cysts at 24 and 48 hours, respectively; the 82 C strain produced 1.08% (24 h) and 3% (48 h); and the 97H strain formed 0.42% (24 h) and 0.67% (48 h) (Fig. 6C). These data indicate that alkaline pH can induce cyst formation across different *T. foetus* strains.

Previously, Iriarte et al. reported that *T. foetus* incubated under nutritional stress conditions increases its DNA content and subsequently generates numerous parasites by multiple fission when favorable external conditions are restored, as a survival strategy associated with a subsequent exponential multiplication of these protozoa^17^. Considering this, we evaluated whether exposure to pH 8 for 24 and 48 hours alters the DNA content of *T. foetus* cysts (strains K, 82 C, and 97H). Parasites were fixed, stained with propidium iodide (PI), and analyzed by flow cytometry to assess DNA content. As a control, the DNA content of *T. foetus* parasites grown in standard TYM culture medium was measured. Parasites incubated at pH 8 exhibited more than a twofold increase in DNA content compared with controls at both 24 and 48 hours in strains K, 82C and 97H  (Fig. 6D, E and Supplementary Data 1). Additionally, multinucleated parasites were  observed in all treatments (Fig. 6F). Under pH 8 exposure, the K strain showed 50.37%, 31.77% and 18.13% of parasites with one nucleus (1 N), two nuclei (2 N) and more than two nuclei (+2 N), respectively, at 24 h; and 49.90%, 25.77% and 24.37% for the same categories at 48 h. The 82 C strain displayed 71.90%, 23.27% and 5.20% of 1 N, 2 N and +2 N parasites, respectively, at 24 h, and 74.50%, 18.00% and 7.50% at 48 h. For the 97H strain, the proportions at 24 h were 71.73% (1 N), 19.15% (2 N) and 9.00% (+2 N), and at 48 h were 72.50%, 16.50% and 11.00%, respectively (Supplementary Fig. 2).

In conclusion, exposure to alkaline pH is associated with the formation of cyst-like structures in multiple *T. foetus* strains, together with increased DNA content and the presence of multinucleated forms. Collectively, these observations suggest that alkaline conditions may contribute to cyst formation and parasite survival, potentially influencing the epidemiology and transmission of bovine trichomonosis.

**Fig. 7:**
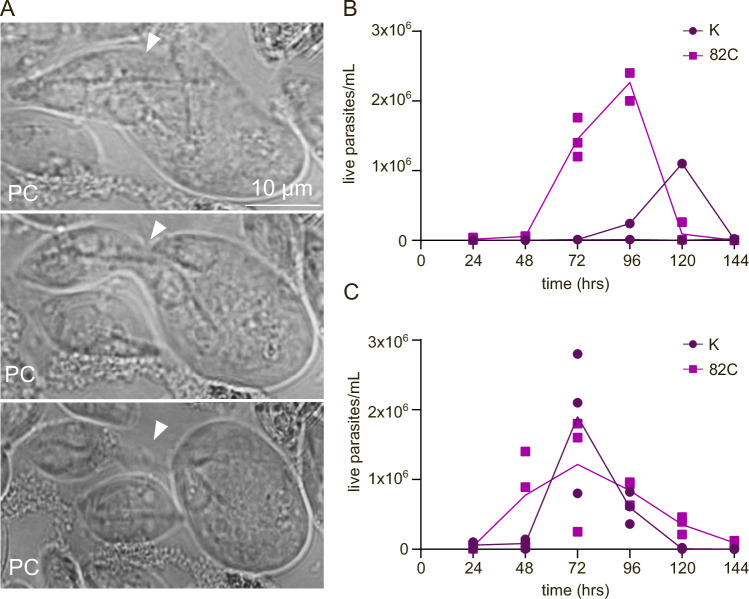
Excystment and growth recoveryof T. foetus after alkaline-induced encystment. **A** Representative image of excystment of *T. foetus* K strain after restoration of standard culture conditions following encystment induced by incubation in TYM at pH 8 for 48 hours. The white arrow indicates the scission area. PC, phase-contrast image. Scale bar, 10 μm. **B**, **C** Growth kinetics of excysted *T. foetus* parasites in TYM medium following 24 hours (**B**) and 48 hours **C** of encystment induction in TYM at pH 8. All individual data points are shown; and lines represent the mean of three independent biological replicates, quantified every 24 h. Source data are provided as a Source Data file.

We next examined the excystment capacity of induced cysts under alkaline conditions. Parasites from K and 82 C strains maintained for 24 h and 48 hours in alkaline culture medium were treated with sarkosyl to isolate resistant forms and then transferred to standard TYM culture medium (pH 6.4). We could observe that both strains were capable of excysting and growing after previous incubation for 24 h and 48 hours at pH 8 (Fig. 7A–C).

Finally, to further investigate cyst resilience, we assessed the survival of *T. foetus* cysts under desiccation and acidic pH, as both represent relevant stressors that cysts may encounter in the environment or following ingestion  by cattle. In the bovine abomasum the ingesta remains for ~3 h at a pH of 2.5. Therefore, we incubated cysts for 3 hours at pH 2.5 and for 7 days under desiccation conditions.Cyst viability was then evaluated by propidium iodide staining. We found that 23% of cysts remained viable after 3 h at pH 2.5, whereas 99% survived desiccation, indicating  that cyst formation markedly enhances *T. foetus* tolerance to harsh environmental and physiological stressors. These findings are preliminary and warrant further in-depth investigation.

### *T. foetus* encystment process transcriptional analysis

**Fig. 8:**
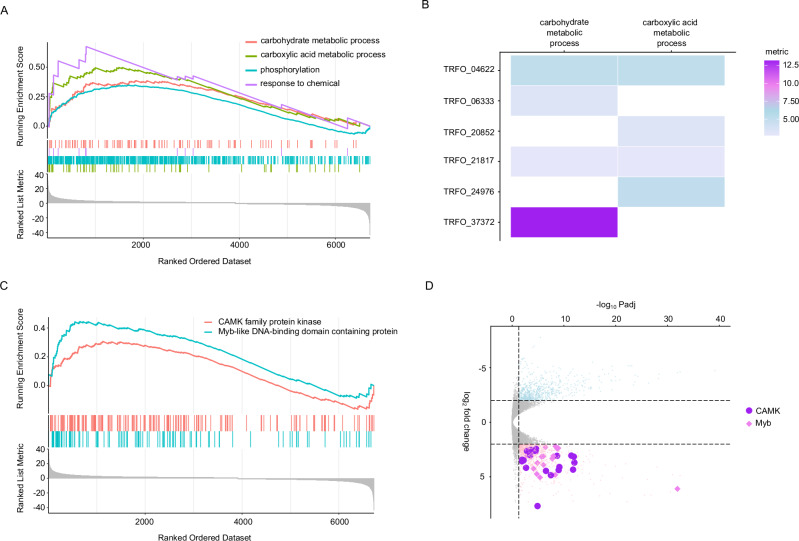
Gene Set Enrichment Analysis of biological processes in *T. foetus* (K strain) under nutritional stress conditions. **A** Enrichment plots for gene sets related to active biological processes. Top: Running enrichment score, representing the cumulative score calculated for each defined gene set (biological process). Middle: Tick marks indicating the rank (position) of each gene belonging to a specific biological process (gene set) within the complete, ranked gene list. Bottom: Ranked list metric, corresponding to the metric used to rank the entire gene dataset. The ranked list represents all analyzed genes, ordered from left to right. Gray bars provide a schematic representation of the metric value for each gene in the ordered list. **B** A heatmap representation of selected leading genes related to N-acetyl-D-glucosamine production (MetaCyc: PWY-7332). The biological processes associated with each gene show carboxylic acid metabolic process (GO:0019752) and carbohydrate metabolic process (GO:0005975). The scale of colors represents values (metric) from the pre-ranked gene set constructed for the GSEA analysis (Methods section). Plotted gene IDs: TRFO_37372 (aldose 1-epimerase), TRFO_24976 (UDP-glucose 6-dehydrogenase), TRFO_21817 (malate dehydrogenase), TRFO_20852 (UDP-glucose 6-dehydrogenase), TRFO_04622 (N-acylglucosamine 2-epimerase), and TRFO_06333 (UDP-glucose 4-epimerase). **C** Enrichment plot for Myb and CAMK kinase families. Top: Running enrichment score, representing the cumulative score calculated for each defined gene set (Myb and CAMK families). Middle: Tick marks indicating the rank (position) of each gene belonging to a specific gene family (gene set) within the complete, ranked gene list. Bottom: Ranked list metric, corresponding to the metric used to rank the entire gene dataset (log2fc) x (-log10pvalue). The ranked list represents all analyzed genes, ordered from left to right according to the ranked list metric. Gray bars provide a schematic representation of the metric value for each gene in the ordered list. (D) Volcano plot of differential  gene expression between unstressed and stressed conditions. Genes with log2 fc >2 or log2 fc < −2 (padj <0.05) are highlighted (light blue = untreated and pink = treated). Myb-like DNA-binding proteins (violet) and CAMK family protein kinase (purple) are highlighted. Source data are provided as a Source Data file.

To evaluate the gene expression profiles associated with encystation in *T. foetu*s, we performed RNA-seq analysis on parasites incubated under nutrient restriction conditions for 48 hours. Gene set enrichment analysis (GSEA) was then applied to identify biological processes significantly enriched under stress conditions. The most enriched processes were carboxylic acid metabolic process (GO: 0019752), phosphorylation (GO: 0016310), response to chemical (GO: 0042221), and carbohydrate metabolic process (GO: 0005975)  (Fig. 8A). These findings are consistent with previous reports in other cyst-forming protozoa, including *Giardia sp*. and *Entamoeba sp*.^16^. Given that these enriched pathways are functionally related to cyst wall biosynthesis, such as N-acetyl-D-glucosamine^16^, we further analyzed the leading genes for each enriched process. Figure 8B highlights six genes involved in the N-acetyl-D-glucosamine biosynthetic pathway, including the UDP-glucose 4-epimerase (TRFO_06333), a relevant enzyme in *Giardia sp*. cyst formation^30^. Next, we performed GSEA across all detected genes in our dataset to identify  enriched protein families under stress conditions. This analysis identified two major protein families enriched under stress conditions: Myb-like DNA- binding proteins and CAMK kinases (Fig. 8C). The enrichment of CAMKs aligns with the previously identified phosphorylation process. Interestingly, Myb-like proteins were not captured in the initial GO enrichment due to the absence of annotated biological processes associated with these genes in the *T. foetus* genome. We then carried out differential expression analysis, which  indicated that these two protein families were the most frequently found (Fig. 8D). These results were consistent with previous reports in *Giardia sp*., where Myb-like proteins and phosphorylation-related proteins were proposed as regulators of the encystation process based on  differential gene expression analysis^31^.

Subsequently, we performed an in silico screening of curated databases (TrichDB.org, AmoebaDB.org, and GiardiaDB.org) to identify candidate enzymes involved in chitin biosynthesis and degradation, as well as enzymes implicated in the formation of the β(1-3)-N-acetyl-D-galactosamine polymer. Components of the chitin synthesis pathway were identified, with the notable exception of chitin synthase. In contrast, all components of the chitin degradation pathway were detected, including a chitinase gene. Furthermore, a complete biosynthetic pathway for the β(1-3)-N-acetyl-D-galactosamine polymer was identified in *T. foetus*, comparable to that described in *Giardia spp.* (Supplementary Fig. 3). Importantly, candidate genes for these pathways were supported  by our RNA-seq analysis (Supplementary Data 2).

### Monosaccharide analysis reveals a HexNAc-rich cyst wall composition

To provide direct biochemical support for the transcriptomic and in silico predictions, we next analyzed the monosaccharide composition of cyst wall-enriched preparations.

Acid hydrolysis followed by HPAEC-PAD analysis of cyst wall-enriched preparations revealed a monosaccharide composition dominated by amino sugars and neutral hexoses (Table 1). Glucosamine and galactosamine were consistently detected as major components in both independent samples, together with mannose, glucose and galactose, while only trace amounts of fucose were observed. Notably, no acidic sugars, including glucuronic or galacturonic acids, were detected in any of the samples. The predominance of glucosamine and galactosamine is consistent with the presence of polymers composed of N-acetylated hexosamines, supporting the existence of a rigid, polysaccharide-rich cyst wall. These biochemical findings independently corroborate the microscopy-based detection of β-linked glycans, and the transcriptomic enrichment of carbohydrate biosynthesis pathways observed in cysts formed under stress conditions.

**Table 1:** Monosaccharide composition of cyst wall-enriched preparations from *T. foetus* cysts

Monosaccharide	Relative abundance (Sample 1)	Relative abundance (Sample 2)
Galactosamine (GalNH₂)	1.11	1.13
Glucosamine (GlcNH₂)	1.89	1.92
Galactose (Gal)	1.00	1.00
Glucose (Glc)	2.66	2.30
Mannose (Man)	3.29	3.12
Fucose (Fuc)	traces	traces
Uronic acids (GlcA, GalA)	not detected	not detected

Lyophilized cyst wall preparations were subjected to acid hydrolysis with trifluoroacetic acid (TFA), and released monosaccharides were analyzed by high-performance anion exchange chromatography with pulsed amperometric detection (HPAEC-PAD). Relative abundances are expressed as peak area ratios normalized to galactose (Gal = 1.00). Two independent samples were analyzed. Uronic acids were below the detection limit in all samples.

### Metronidazole treatment induces *T. foetus* encystation

**Fig. 9:**
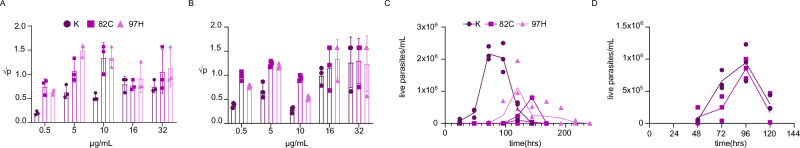
Metronidazole induces cyst formation in *T. foetus.* Quantification of sarkosyl-resistant parasites after incubation in TYM medium with increasing concentrations of metronidazole (0.5, 5, 10, 16, and 32 µg/ml) in different *T. foetus* strains (K, 82 C, 97H) for 24 hours **A** and 48 hours **B**. Data represent the percentage of sarkosyl-resistant parasites. Proportions were arcsine square-root transformed prior to statistical analysis. Data are presented as mean ± s.d. of the transformed values (n = 3 biological replicates). Percentages are provided in the main text for clarity and biological interpretation. Growth kinetics of excysted *T. foetus* parasites in TYM medium following 24 hours **C** and 48 hours **D** of encystment induction by incubation in TYM containing 10 µg/ml. All individual data points are shown; and lines represent the mean of three independent biological replicates. Parasites were quantified every 24 hours. Source data are provided as a Source Data file.

Historically, the treatment of bovine trichomonosis relied on 5-nitroimidazole compounds, such as metronidazole or 1- (2-hydroxyethyl)-2- methyl-5-nitroimidazole. However, trichomonads have been reported to develop resistance to metronidazole under specific conditions^32,33^. Interestingly, previous studies demonstrated that pseudocysts represent a reversible form induced by several drug treatments^7,12^. Therefore, we determined whether metronidazole treatment induces the encystation  process in *T. foetus*. First, we evaluated the presence of true cysts in parasites treated with different drug concentrations. *T. foetus* trophozoites from K, 82 C, and 97H strains were incubated in TYM culture medium supplemented with 0.5, 5, 10, 16 and 32 µg/ml of metronidazole for 24 h and 48 hours. Samples were then centrifuged and exposed to 0.15% sarkosyl to lyse the trophozoites. After 24 hours of treatment, 3.74%, 35%, 25.38%, 51.1% and 45.83% of parasites formed  sarkosyl-resistant structures in the *T. foetus* K strain at 0.5, 5, 10, 16 and 32 µg/ml of metronidazole, respectively. In the 82 C strain, 46.3%, 74.64%, 89.83%, 48.3% and 67.6% cysts were  observed at the same drug concentrations . Similarly, 34.36%, 98.33%, 92.21%, 60.66% and 74.53% of 97H parasites corresponded to  true cysts at 0.5, 5, 10, 16 and 32 µg/ml of metronidazole, respectively. After 48 h of treatment, 16.66%, 36.64%, 8.16%, 69.43% and 79.33% of parasites formed  sarkosyl-resistant structures in *T. foetus* K strain at 0.5, 5, 10, 16 and 32 µg/ml of metronidazole, respectively. In the 82 C strain, 73.70%, 91.21%, 67.91%, 75.83% and 82.33% cysts were observed at the same drug concentrations. For the 97H strain, 48.81%, 87.5%, 27.5%, 86.66% and 76.33% of parasites corresponded to  true cysts at 0.5, 5, 10, 16 and 32 µg/ml of metronidazole, respectively (Fig. 9A, B). We then evaluated whether  metronidazole-induced cysts were capable of reverting to trophozoites. First, *T. foetus* trophozoites from K, 82 C, and 97H strains were incubated in TYM culture medium with 10 µg/ml of metronidazole for 24 and 48 hours.  Sarkosyl-resistant parasites were subsequently transferred to  fresh culture medium to asses  growth recovery . K, 82 C, and 97H strains reverted to trophozoites after 24 hours of metronidazole exposure , whereas  only K and 82 C strains were able to revert  after 48 hours of treatment  (Fig. 9C, D).

Then, we estimated the DNA content of parasites treated with metronidazole (10 µg/ml) for 24 hours. The K strain (control) showed 67.66%, 21.83%, 7.85% and 2.63% of cells with 1 C, 2 C, 3-4 C and >4 C nuclear DNA content, respectively. The K strain incubated in TYM culture medium with 10 µg/ml of metronidazole for 24 hours exhibited 11.45%, 6.275%, 18.35 % and 63.95% of parasites with 1 C, 2 C, 3-4 C, and >4 C nuclear DNA content, respectively. 82 C strain (control) presented 71.25%, 20.33%, 6.76%, and 1.37% of parasites with 1 C, 2 C, 3-4 C and >4 C nuclear DNA content, respectively. The 82 C strain incubated in TYM with 10 µg/ml of metronidazole for 24 h exhibited 57.7%, 20.7%, 13%, and 8% of parasites with 1 C, 2 C, 3-4 C, and >4 C nuclear DNA content, respectively. Finally, the 97H strain (control) showed 73.33%, 12.99%, 9.83% and 1.55% of parasites with 1 C, 2 C, 3-4 C and >4 C nuclear DNA content, respectively. The 97H strain incubated in TYM with 10 µg/ml of metronidazole for 24 hours presented 49.5%, 13.65%, 18.1% and 17.65% of parasites with 1 C, 2 C, 3-4 C, and >4 C nuclear DNA content, respectively (Supplementary Fig. 4). These results indicate that metronidazole treatment is associated with increased DNA content and the formation of true cysts in *T. foetus*. These cysts are capable of reverting to trophozoites, suggesting that cyst formation may represent a resistance mechanism employed by *T. foetus* against metronidazole treatment.

Lastly, to assess whether cyst wall formation contributes to metronidazole resistance in *T. foetus*, trophozoites were incubated with metronidazole either alone or in combination with nikkomycin Z, a competitive analogue of the chitin synthase substrate UDP-N-acetylglucosamine (Supplementary Fig. 3), and parasite viability was subsequently assessed. Trophozoites exposed to metronidazole alone showed a viability of 20.2 ± 1.7%, whereas those treated with both metronidazole and nikkomycin Z exhibited a viability of 0.95 ± 0.55%. These results support the involvement  of the predicted biosynthetic pathways and indicate that parasites were significantly more susceptible to metronidazole when cyst wall synthesis was impaired.

## Discussion

Cyst formation is considered an adaptive strategy employed by different microorganisms to withstand adverse environmental conditions. This process contributes to long-term survival and cell dispersal^34^. Among protozoa, ciliates represent a prominent group in which cyst formation has been extensively documented. To date, cysts have been described in approximately 40 ciliate species^27^, which showed different morphologies^35^ and sizes, ranging from 15 µm in *Pseudocohnilembus pusillus* to 225 µm in *Blepharisma japonicum*^36,37^. Curiously, the ciliate *Pseudourostyla levis* forms both cysts and pseudocysts (without a true cyst wall), structures that appear to represent  a short-term adaptation to the environment^38^. Beyond ciliates, several free-living protists also form cysts as a survival strategy. Examples include *Acanthamoeba spp*., *Balamuthia mandrillaris*, and *Naegleria fowleri*, all of which propagate independently in the environment and produce disease upon accidental human exposure^39^. In particular, *Acanthamoeba castellanii* can reversibly differentiate into either cysts or pseudocysts, depending on the nature and duration of environmental stress. Both resting stages are characterized by distinct cell surface: a double-layered wall in cyst^40,41^ and a single-layered fibrillar coat on the pseudocyst^42^. Interestingly, Acanthamoeba cysts induced by desiccation have demonstrated viability and pathogenic potential after more than two decades^43^, highlighting the extreme resilience of these structures. Alternatively, cyst formation in some parasitic protists takes place within the host. Examples include *Toxoplasma gondii*, *Giardia lamblia*, *Entamoeba histolytica*, *Cryptosporidium parvum*, and *Balantidium coli*. These parasites differ from free-living protists in that their cysts function primarily in host-to-host transmission.

The parabasalids are a group of flagellated anaerobic protists that have evolved as symbionts of the animal digestive tract, causing little or no harm to their hosts. However, some species have adapted to colonize other areas of the body, where they behave as parasites^44^. Their life cycle generally involves only free-swimming trophozoites, and the presence of true cysts has been reported only in a limited number of species^13,14^. Specifically, *Monocercomonas ruminantium* and *Honigbergiella ruminantium*, inhabitants of the bovine gastrointestinal tract, form true cysts^45^. Remarkably, *Honigbergiella sp*. is a commensal able to survive for certain periods in freshwater. These protists have been isolated from bull preputial samples and a creek contaminated by cattle feces, demonstrating a great adaptation capacity to different environmental conditions^14^. Several parabasalids form pseudocysts, which are thought to contribute to environmental survival. In *Trichomonas gallinae*, a parasite that infects the upper digestive tract in columbiformes, falconiforms, raptors, and passeriforms, pseudocysts have been proposed as a resistant form of parasite survival during fecal-oral transmission^8,9^. Similarly, *Tetratrichomonas gallinarum*, found in the intestinal tract of poultry and transmitted through contaminated feed or drinking water^44^, relies on pseudocyst formation for environmental persistence^9^. Beri et al. reported that *T. vaginalis* forms viable cyst-like structures containing wall-associated polysaccharide deposits detected by CFW staining and exhibiting resistance to osmotic and detergent stress, features that closely parallel the findings obtained in our study for *T. foetus*^46,47^, Pseudocysts have likewise been described in *Tritrichomonas muris*, a rodent gut colonizer, and in *Trichomonas tenax*, a commensal^48^. In *T. muris*, fecal-oral transmission occurs via ingestion of either the pseudocyst or trophozoites^49^. Recently, we demonstrated that *T. foetus* can survive passage through the gastrointestinal tract in bovines, be discharged by feces, and contaminate the cow’s reproductive tract, likely due to their anatomical position. The parasite survives a range of pH conditions typically found in the bovine gastrointestinal tract and persists in bovine feces and water for several days^3^. In cats, *T. foetus* infection occurs predominantly via the fecal-oral route, and the parasite remains viable in cat feces and wet cat food for several days^50,51^. *T. foetus* forms pseudocysts in response to environmental stressors, such as nutrient deprivation, exposure to specific drugs, or abrupt temperature shifts, serving as a survival strategy^7,12^. In this study, we demonstrated that *T. foetus* forms true cysts under nutritional stress. These cysts possess a defined wall enriched in specific polysaccharides, consistent with a previous report that identified the presence of chitin on the *T. foetus* cell surface using anti-chitin antibodies^52^. We further showed that *T. foetus* cysts were positive to WGA staining, a lectin that specifically binds to N-acetyl-D-glucosamine (found in chitin) and N-acetyl-D-neuraminic (sialic) acid residues. Consistent with this,  we identified genes related to the UDP-N-acetylglucosamine synthesis pathway upregulated during the encystation process. Among these, a UDP-glucose 4-epimerase, a key enzyme involved in cyst wall biosynthesis in *Giardia*^53^ and *E. histolytica*^54^, was identified here.

Although chitin synthase could not be identified in the current in silico analysis of the *T. foetus* genome, the presence of chitin-like polysaccharides is supported by fluorescence staining and monosaccharide composition analysis. The absence of a canonical chitin synthase may reflect three possibilities: (1) the high sequence divergence of this enzyme in early-branching protists, (2) limitations in current genome annotation approaches, or (3) that this function is carried out by a complex multi-protein system^55^. Notably, several enzymes associated with chitin and hexosamine turnover, including chitinases and β-N-acetylhexosaminidases, were identified and transcriptionally active under encystation conditions. This observation suggests that, in addition to polysaccharide synthesis, extensive remodeling and recycling of hexosamine-containing polymers may occur during cyst formation and potentially during excystation, a feature that could contribute to the dynamic and reversible nature of the cyst wall. Our findings suggest that the cyst wall of *T. foetus* may display a higher degree of chemical complexity than those described in other well-characterized cyst-forming protozoa. While the cyst wall of *Giardia spp*. is largely composed of a homopolymer of N-acetylgalactosamine associated with cyst wall proteins, and that of *Entamoeba spp*. is predominantly chitin-based, monosaccharide analysis of *T. foetus* cyst wall-enriched preparations revealed the presence of both glucosamine and galactosamine. Together with the detection of β-linked polysaccharides by fluorescence staining and the transcriptomic enrichment of carbohydrate biosynthesis pathways, these findings are consistent with a cyst wall architecture incorporating multiple N-acetylated hexosamine-containing polymers.

Our transcriptomic analysis also revealed enrichment in two major gene families: Myb-like DNA-binding proteins and CAMK kinases. In *Giardia*, the Myb2 transcription factor has been proposed as a master regulator of encystation^56^. In *Entamoeba sp*., a Myb transcription factor modulates the expression of several cyst-specific genes^57^, including chitin synthase, chitinase, putative kinase, Fe-hydrogenase, and developmentally regulated proteins. Similarly, the Myb-like transcription factor (BFD1) in *T. gondii* is required for differentiation into chronic cysts-resistant stage^58^. We also identified a malate dehydrogenase (TRFO_21817) gene differentially expressed during encystation. Interestingly, this gene has been associated to the N-acetyl-glucosamine synthesis process and contains a putative Myb-binding site in its promoter^59^. Structural similarities between *T. foetus* and other protists further underscore the conserved nature of cyst wall biogenesis. In *E. invadens*, the cyst wall is mainly composed of chitin, a β-(1,4)-linked homopolymer of N-acetyl-D-glucosamine^16^. The presence of glycoconjugates has been demonstrated in the cyst wall of some ciliates^60,61^. Chessa et al.^62^ showed that cyst maturation in *Colpoda cucullus* involves compaction of layers, deposition of new material on the inner side of the cyst wall and cytochemical rearrangements of its glycoprotein components.

One of the most remarkable changes during cyst formation is a drastic decrease in cellular volume; achieved through autophagy, cytoplasmic dehydration, metabolic inactivation, ciliature resorption/regeneration, and the assembly of a resistant but permeable cyst wall^15,26^. The final size probably reflects an optimum balance between minimizing the cell volume and preserving sufficient biological material to undergo excystment. In *T. foetus*, we observed flagellar internalization, cytoplasmic reorganization and the absence of detectable cytoskeletal staining during encystation, supporting the idea of cellular simplification during the process. Similarly, the pseudocyst stage in *T. foetus* is characterized by the regression or loss of key cytoskeletal structures, including the costa, flagella, and the undulating membrane^12^. Comparable cytoskeletal remodeling occurs in other protists during encystation. For instance, encystation in *E. histolytica*, *E. invadens*, and *A. castellanii*, is coupled to major changes in the cytoskeleton, gene expression, protein transport, metabolism and nuclear division^16^. These nuclear changes result in a tetranucleated structure, formed through two successive rounds of chromosome replication without cell division^16^. Similarly, we demonstrate here that DNA endoreplication occurs in *T. foetus* cyst structures.

Our in vitro assays revealed that cyst induction in *T. foetus* occurs under alkaline conditions (pH 8), similar to those  of preputial mucus in bulls, whereas excystation is triggered when the environment shifts to pH 6.4, which mimics the vaginal mucus of cows. These findings align with existing evidence that various parasites form cysts in response to environmental stressors such as nutrient depletion, pH shifts, osmotic stress, or exposure to compounds like bile and hydrogen peroxide^16^. Moreover, our results show that *T. foetus* increases its DNA content during incubation under alkaline conditions, suggesting that cysts may form in the alkaline preputial environment of bulls while undergoing DNA endoreplication, and subsequently excyst by multiple fission when optimal conditions are restored (e.g., in the cow reproductive tract at pH 6.5-7). Thus, bulls could transmit cystic structures with the potential to generate multiple trophozoites in the reproductive system of cows. These findings provide newl insights into the biology of *T. foetus* and may have important implications for understanding the epidemiology and transmission dynamics of this parasitic infection in bovines.

Finally, the standard treatment for bovine trichomonosis has historically relied on metronidazole and other related 5-nitroimidazole derivatives. However, resistance to metronidazole has been increasingly reported in *T. foetus*, and the use of nitroimidazoles has been prohibited in food-producing animals^33,63^. Our findings indicate that *T. foetus* may resist metronidazole through cyst formation, pointing to a previously unrecognized resistance mechanism. This observation is consistent with the fact that metronidazole is a relatively small, lipophilic molecule that enters cells by passive diffusion to reach its intracellular targets^64^. Moreover, our results indicate that cell wall formation contributes to this resistance, as co-treatment with nikkomycin Z, a competitive analogue of UDP-N-acetylglucosamine that inhibits the cell wall biosynthesis pathway, markedly reduced the ability of parasites to withstand metronidazole treatment. This discovery raises new challenges for the development of therapeutic alternatives and highlights the urgent need to better understand the encystation/excystation processes in *T. foetus* to minimize its economic impact on the livestock industry.

## Methods

### Parasites cultures

*T. foetus* K strain (Embrapa, Rio de Janeiro, Brazil) and 82 C and 97H strains (Buenos Aires, Argentina) were cultured in Diamond’s trypticase-yeast extract-maltose (TYM) medium supplemented with 10% horse serum and 10 U/ml penicillin/10 µg/ml streptomycin (Invitrogen) at pH 6.4. Parasites were grown at 37 °C and passaged daily. 1 N NaOH and 5 N HCL were used to adjust the pH of the medium to the desired value.

### Encystment assay

To induce *T. foetus* cyst formation, strains K, 82 C, and 97H were incubated under the following conditions: (A) Nutrient deprivation: Parasites (1 × 10^6^ parasites/ml) were incubated in TYM culture medium without maltose and serum supplementation for 48 or 96 hours. (B) Alkaline pH: 1 × 10^6^ parasites/ml were incubated in TYM medium adjusted to pH 8 for 24 or 48 hours. (C) Metronidazole induction: Parasites (1 × 10^6^ cells/ml) were incubated in TYM medium supplemented with 0.5, 5, 10, 16, and 32 µg/ml of metronidazole for 24 or 48 hours at 37 °C. Samples were collected at 48 and 96 hours for the nutrient deprivation condition and at 24 and 48 hours for alkaline pH and metronidazole treatments. Following incubation, samples were centrifuged and treated with 0.15% sarkosyl for 15 min at 28 °C to lyse trophozoites. Encystment percentage was calculated as the number of sarkosyl-resistant cells divided by the total number of *T. foetus* initially subjected to the induction process.

### Excystment assay

To evaluate the ability of cyst structures to revert to trophozoites, an excystment assay was performed. Following the encystment process, cysts were washed to remove residual sarkosyl and resuspended in fresh TYM medium supplemented with equine serum to promote the reversion. Samples were incubated at 37 °C, and aliquots were taken at different time points to monitor excystment by assessing trophozoite motility under a microscope. The number of trophozoites was quantified using a Neubauer chamber.

### Parasite growth assay

Growth curves were assessed using different *T. foetus* strain (K, 82 C, and 97H). For these experiments, cysts were inoculated in 10 ml of Diamond’s medium and incubated at 37 °C. Cell counts were recorded using a hemocytometer. Parasite counts were recorded at the indicated times on the x-axis. Results represent the average of three independent experiments, and error bars represent s.d.

### Fluorescent staining

(A) Calcofluor White staining: parasites were incubated with 0.01% Calcofluor White stain (comprising 1 g/l CFW M2R and 0.5 g/l Evans blue) (Sigma-Aldrich) in PBS (pH 7.2) for 30 min at 26 °C.

(B) Wheat germ agglutinin: cysts or trophozoites were incubated for 1 h at 37 °C in a 1:100 dilution of FITC-conjugated lectin from *Triticum vulgaris* (WGA, Sigma-Aldrich)

(C) Congo red staining: cysts or trophozoites were stained with a 0.004% Congo red solution for 15 min

(D) Trypan blue staining: cysts or trophozoites were incubated with a 0.4% Trypan blue solution for 5 min.

After staining, slides were washed three times in PBS, mounted on Fluoromont (Sigma-Aldrich), and observed under an inverted fluorescence microscope (Zeiss Axio Observer 7 inverted fluorescence microscope, USA).

(E) Cell Tracker Red staining: *T. foetus* trophozoites were labeled using CellTracker Red CMTPX Dye (ThermoFisher) for 45 min. Parasites were then incubated in TYM culture medium without maltose and serum for 48 hours. Samples were collected, centrifuged, and treated with 0.15% sarkosyl for 15 min at 28 °C to lyse the trophozoites, preserving resistant cyst forms. Samples were analyzed using a Zeiss Axio Observer 7 (Zeiss) microscope.

### Stress assays under acidic and desiccation conditions

To evaluate cyst survival under acidic and desiccation conditions, *T. foetus* trophozoites were induced to encyst by nutritional stress (96 hours in TYM medium without maltose) and enriched by sarkosyl treatment (0.01%, 15 min). Sarkosyl-resistant structures (cysts) were then exposed to pH 2.5 for 3 hours or desiccated on filter paper for 7 days, after which viability was assessed by propidium iodide staining.

### Nikkomycin Z assay

*T. foetus* trophozoites were pre-incubated in TYM medium containing 100 µg/ml nikkomycin Z (MedChem Express) for 3 hours, after which 20 µg/ml metronidazole was added, and the cultures were incubated for 48 hours at 37 °C. In parallel, trophozoites were incubated in TYM medium containing 20 µg/ml metronidazole alone (cyst-induction control). Following incubation, parasite viability was assessed by propidium iodide staining.

### Immunolocalization experiments

Parasites incubated for 48 hours under nutrient-restricted conditions were centrifuged and resuspended in 0.15% sarkosyl for 15 min. After washing with PBS, the cells were incubated on glass coverslips at 37 °C for 2 hours, then fixed and permeabilized in cold methanol for 10 min. Cells were washed and blocked with 5% fetal bovine serum (FBS) in phosphate-buffered saline (PBS) for 30 min and incubated with a 1:500 dilution of anti-TUBA4A (TUBA1) Antibody - Mouse monoclonal, B-5-1-2 (Sigma-Aldrich: T5168). Coverslips were washed with PBS and incubated for 1 hour at room temperature (RT) with a 1:5000 dilution of Goat anti-Mouse IgG (H + L) Cross-Adsorbed Secondary Antibody, Alexa Fluor™ 594. Invitrogen/Thermo Fisher Scientific: Catalog # A-11005. Coverslips were mounted onto microscope slips using ProLong Gold antifade reagent with DAPI or 4,6-diamidino-2-phenylindole (Invitrogen). Observations were performed on a Zeiss Axio Observer 7 (Zeiss) inverted fluorescence microscope. Adobe Photoshop (Adobe Systems) was used for image processing.

### Confocal microscopy analysis

Confocal images were acquired using an IX-81 microscope coupled to an FV-1000 confocal module with a Plan APO 60, 1.42-numerical-aperture (NA) oil immersion objective (Olympus, Japan). Image acquisition was performed using FV 10-ASW 3.1software. Images were processed using ImageJ 1.45 s software.

### Scanning electron microscopy (SEM)

Cells were washed with PBS and fixed in 2.5% glutaraldehyde in 0.1 M cacodylate buffer, pH 7.2. Cells were post-fixed for 15 min in 1% OsO4, dehydrated in ethanol, and critical point dried with liquid CO_2_. Dried cells were coated with gold-palladium to a thickness of 15 nm and observed using a Jeol JSM-5600 scanning electron microscope operating at 15 kV.

### Transmission electron microscopy (TEM)

Samples were fixed in 2.5% glutaraldehyde in 0.1 M phosphate buffer (pH 7.5) for 4 hours,  followed by post-fixation in 1% osmium tetroxide in 0.2 M phosphate buffer (pH 7.5) for 1 h and 30 min. Samples were dehydrated in a graded ethanol series (50%, 70%, 96%, and 100%) followed by acetone. Samples were infiltrated in a resin-acetone mixture (1:2) and embedded in epoxy resin overnight. Then, the samples were oriented in silicone molds and polymerized in an oven at 60 °C for 72 hours. Finally, Ultrathin sections were contrasted with 2% uranyl acetate for 20 min and 2% lead citrate for 3 min. Samples were examined using a Zeiss EM109T transmission electron microscope operating at 80 kV, equipped with a Gatan ES1000W camera.

### Analysis of *T. foetus* in preputial mucus samples

Preputial mucus samples from three bulls were collected and incubated with 1 × 10^5^ trophozoites for 48 hours at 37 °C. Structures were then stained with CFW staining. Observations were performed using a Zeiss Axio Observer 7 (Zeiss) inverted fluorescence microscope. Adobe Photoshop (Adobe Systems) was used for image processing.

### Flow cytometry

To determine DNA content, parasites (5 × 10^6^ cells/ml) were harvested and fixed in 5 ml of ice-cold 100% EtOH at 4 °C overnight. Each sample was washed in 1 ml of PBS containing 2% vol/vol horse serum (HS), resuspended in 1 ml of PBS containing 180 μg/ml RNase A (Sigma- Aldrich, Buenos Aires, Argentina) and 2% vol/vol HS, and incubated for 30 min at 37 °C to digest RNA. Samples were then stained with a 25 μg/ml propidium iodide (PI) and incubated for 30 min at 37 °C prior to flow cytometry analysis. Flow cytometry was performed using a FACS Calibur flow cytometer (Becton Dickinson, San Jose, USA) equipped with a dual laser system (15 mW 488 nm argon ion laser and a 635 nm red diode laser). For DNA content measurement, cells were excited with 480 nm light and emission was collected using a 585/42 filter (PI fluorescence; FL2). Data from 20,000 cells were recorded, and analyzed using the FlowJo 7.6 software. Correlation between light-scattering properties and DNA content was performed by setting electronic gates on forward scatter (FSC) versus side scatter (SSC) profiles and evaluating DNA content within each gate (Supplementary Fig. 5).

### RNA-seq analysis

Total RNA from *T. foetus* parasites (strain K) was extracted using the RNAspin Mini RNA Isolation Kit (GE Healthcare Life Sciences). Three independent RNA samples were obtained from parasites incubated for 48 hours in serum-free culture medium. As a control, RNA was extracted from parasites cultured under standard conditions with serum suplementation. To eliminate genomic DNA contamination, all RNA samples were treated with DNase I (Invitrogen) before amplification. Library preparation and sequencing were carried out by Quick Biology. Samples were sequenced using Illumina sequencers. Reads were demultiplexed using Illumina bcl2fastq2 v2.20 with default settings. Read quality was assessed using FastQC, and approximately 20 million reads were generated per sample.

### Differential Gene Expression Analysis

Quality control of reads for each Illumina library was performed using the FASTQC tool (A Quality Control Tool for High Throughput Sequence Data Online). Adapter trimming and filtering of low-quality reads (length shorter than 35 nucleotides and quality score lower than phred 30) were carried out using the TrimmGalore software (https://github.com/FelixKrueger/TrimGalore). Filtered reads were aligned *T. foetus* strain K1 reference genome (ASM183968v1) using the HISAT2 tool. Transcript quantification was performed following the StringTie protocol, utilizing the alternative step for generating the raw count matrix (script: prepDE.py). The raw count matrix of *T. foetus* genes was used as input for the DESeq2 package in R to perform differential gene expression analysis (adjusted p-value < 0.05 and log2FC ≥ 2). Principal component analysis (PCA) was performed using the DESeq2 package in the R environment (v4.4.2) (Supplementary Fig. 6).

### Gene Set Enrichment Analysis

Results from DESeq2 analysis were used for metric construction as follows: log2fc*-log10(pvalue). This metric was used to construct a sorted, pre-ranked list of all the transcripts detected in the data set. Only transcripts with padj <padj<0.25 were considered for enrichment analysis. GSEA was performed using functions GSEA() and gseGO() from the clusterProfiler package (91). The parameters used for GSEA() analysis were minGSSize = 10, maxGSSize = 600, pvalueCutoff = 0.05, eps = 0, seed = TRUE, and pAdjustMethod = “BH.” For gseGO() analysis, the same parameters, if corresponding, were used except for nPermSimple = 100000. Only biological processes were considered. Results were collapsed by redundant terms using the simplify function (91). Plots were made with the enrichplot package (92). For the heatmap plot, only leading genes from a metabolic pathway related to N-acetyl-glucosamine production were shown (MetaCyc: PWY-7332). All the *T. foetus* genes related to that pathway were obtained from Trichdb.org database using the search Identify Genes based on Metabolic Pathway” with default options except for EC Exact Match Only = No. The resulting list of genes was used to filter the gseGO analysis, and the resulting genes were plotted. The scale values represented in the plot correspond with the pre-ranked list of genes used for the GSEA. All analyses and plots were conducted under an R environment (v4.4.2). Data and metrics used to construct the related plots can be found in Supplementary Data 3.

### Bioinformatic prediction of cyst wall biosynthesis

Information on enzymes and EC number involved in chitin synthesis and β(1-3)-N-acetyl-D-galactosamine polymer biosynthesis was retrieved from a previously published study^65^. Annotated genes associated with each EC numbers were obtained from Trichdb.org: *Tritrichomonas foetus*, *Trichomonas vaginalis*, *Histomonas meleagridis*; Amoebadb.org: *Dictyostelium spp*., *Acanthamoeba castellanii*, *Entamoeba spp*., *Mastigamoeba balamuthi* and *Naegleria gruberi*; Giardiadb.org: *Giardia spp*., *Monocercomonoides exilis* and *Spironucleus salmonicida*. A summary of the genes detected in *T. foetus* by RNA-seq analysis is presented in Supplementary Data 2.

### Enrichment and sugar analysis of the cyst wall of *T. foetus*

To analyze the sugar composition of the cyst wall, 5 × 10⁸ *T. foetus* parasites were induced to encyst under nutritional stress conditions. After 96 hours, parasites were harvested and washed once with ice-cold phosphate-buffered saline (PBS). Cells were disrupted by five freeze-thaw cycles, consisting of freezing for ≥2 min in liquid nitrogen followed by thawing for 5 min at 37 °C with gentle vortexing. The lysate was centrifuged at 5,000 × g for 10 min at 4 °C, and the resulting pellet was resuspended in 1 ml of PBS. An equal volume of phenol:chloroform:isoamyl alcohol was added, and samples were vortexed for 60 s. Phase separation was achieved by centrifugation at 12,000 × g for 10 min at 4 °C, and the interface fraction was carefully collected . Three volumes of ice-cold absolute ethanol (-20 °C) were added, and samples were briefly vortexed and incubated for 30 min at -20 °C to allow precipitation. Samples were centrifuged at 5,000 × g for 10 min at 4 °C. The pellet was washed once with ice-cold ultrapure water, centrifuged at 5,000 × g for 5 min at 4 °C, frozen at -80 °C, and lyophilized to complete dryness. Dried samples were stored at -80 °C until further analysis.

### Monosaccharide composition analysis of cyst wall preparations

Lyophilized cyst wall-enriched pellets were resuspended in ultrapure water and subjected to acid hydrolysis using 2 N trifluoroacetic acid (TFA). Hydrolysis was performed at 100 °C during 3 hours to release constituent monosaccharides. After hydrolysis, samples were dried and resuspended in water prior to analysis.

Monosaccharide composition was determined by high-performance anion exchange chromatography with pulsed amperometric detection (HPAEC-PAD) at the Carbohydrate Analysis Service, Faculty of Exact and Natural Sciences, University of Buenos Aires (UBA), using a Dionex Bio-LC DX-3000 system equipped with a CarboPac P20 analytical column and guard column. Neutral and amino sugars were analyzed under isocratic elution with sodium hydroxide as the mobile phase. Acidic sugars were analyzed separately using sodium acetate gradients.

Identification of monosaccharides was performed by comparison of retention times with authentic standards, including glucosamine, galactosamine, glucose, galactose, mannose, fucose, and uronic acids.

### Reporting summary

Further information on research design is available in the Nature Portfolio Reporting Summary linked to this article.

## Supplementary information

Supplementary Information

Description of Additional Supplementary Files

Supplementary Data 1

Supplementary Data 2

Supplementary Data 3

Reporting Summary

Transparent Peer Review file

## Source data

Source Data

## Data Availability

The RNAseq data generated in this study have been deposited in the GEO database under accession code GSE306990. Source data are provided with this paper.

## Code availability

Custom scripts to reproduce the analyses presented in this work are available on Zenodo at https://zenodo.org/records/18941423.
