# Supplementary material for: True cyst formation underlies persistence and drug tolerance in Tritrichomonas foetus
Source: Nat Commun. 2026 Apr 28;17:5831. doi: 10.1038/s41467-026-71827-9 (PMC13332039; doi:10.1038/s41467-026-71827-9)
Supplement: Supplementary file 1 — Supplementary Information [file 41467_2026_71827_MOESM1_ESM.pdf]

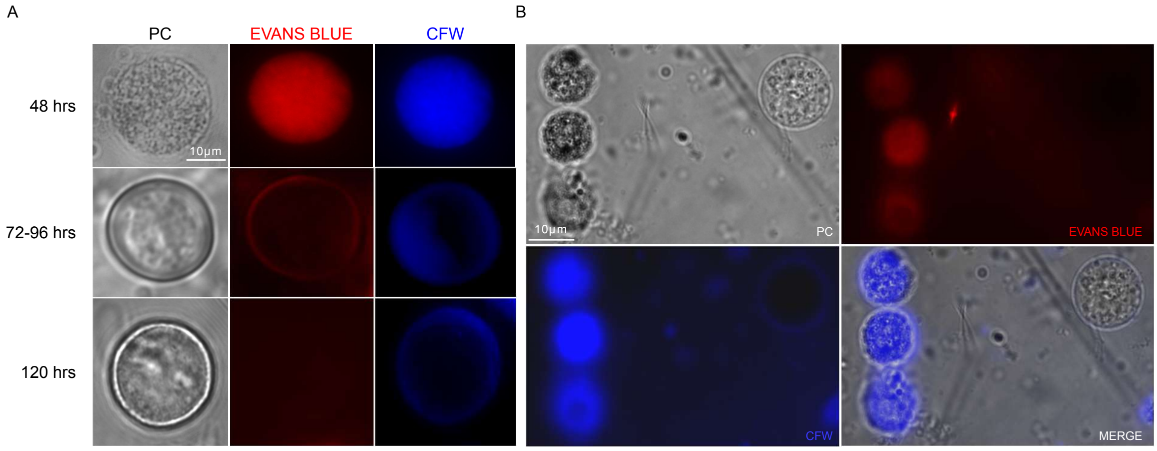

Supplementary Figure 1: Differentiation of parasites during *T. foetus* encystation. (A) Under encystation-inducing conditions, the predominant parasite populations stained with Evans Blue (EB) and/or calcofluor white (CFW) at the indicated time points are shown by fluorescence microscopy. Scale bar, 10 µm. (B) Representative images showing differential staining of parasite populations with EB and/or CFW, visualized by fluorescence microscopy. Images are representative of three independent biological experiments.

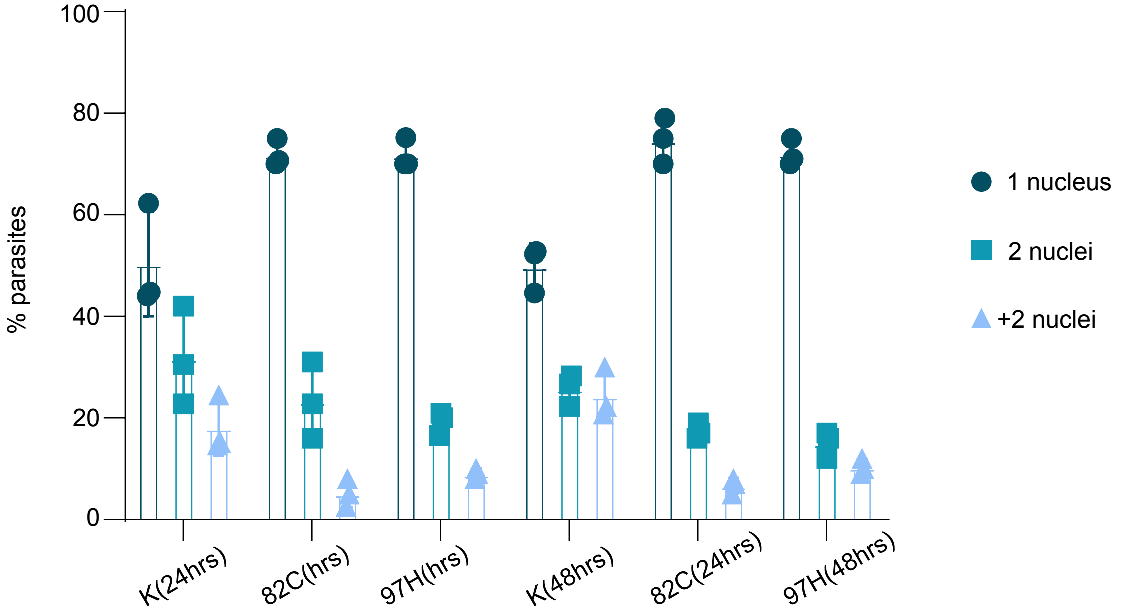

Supplementary Figure 2: Heterogeneity in the number of nuclei in *T. foetus* under alkaline pH conditions. The percentages of parasites with one, two, or more than two nuclei are shown. Different *T. foetus* strains (K, 97H, and 82C) were analyzed after incubation for 24 and 48 h at pH 8. Nuclei were stained with DAPI and examined by fluorescence microscopy. Each point represents an independent biological experiment in which 100 parasites were counted (n= 3 independent experiments). Data are presented as mean ± s.e.m. Source data are provided as a Source Data file.

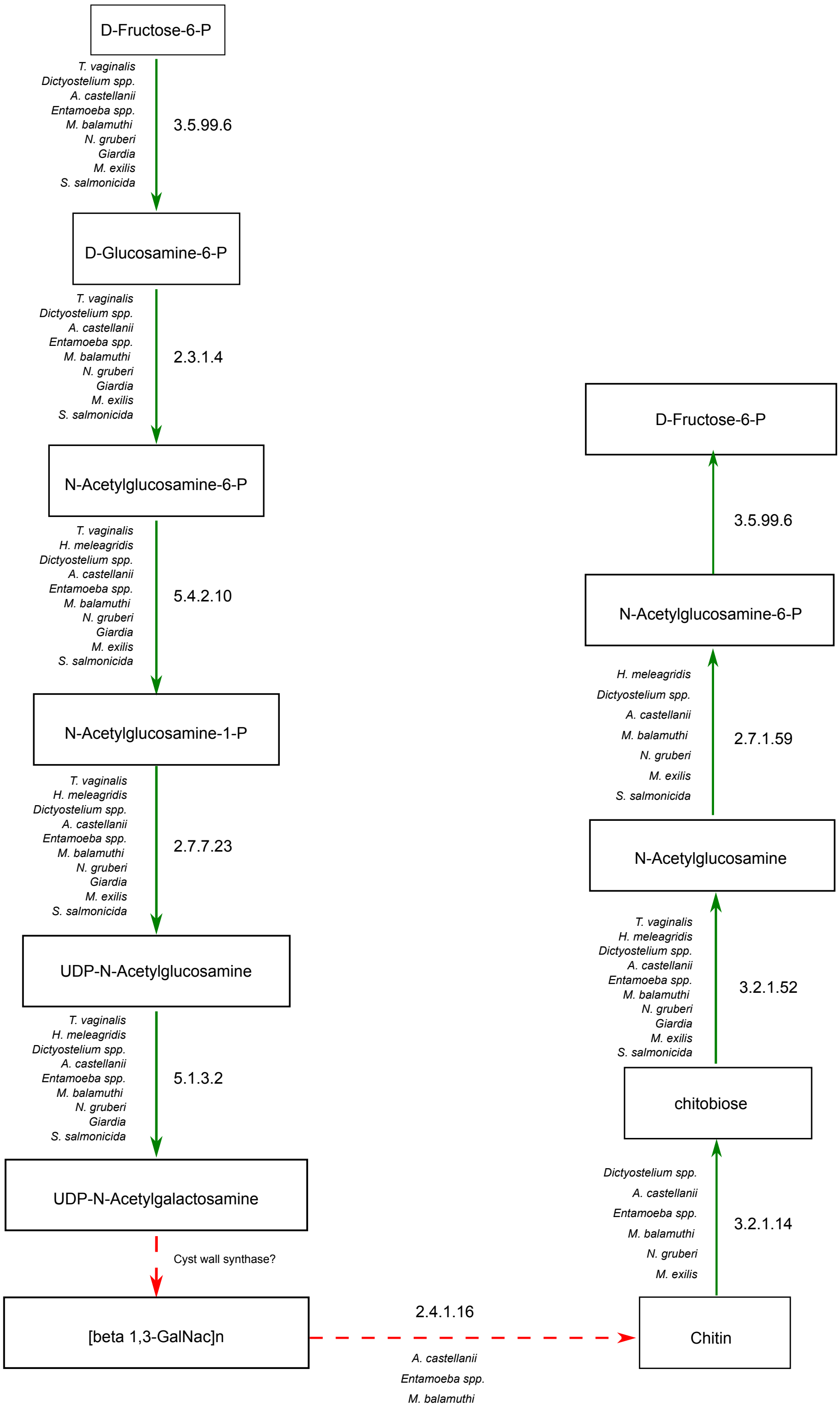

Supplementary Figure 3: Proposed cyst formation and degradation pathway in *T. foetus*. Schematic representation of the biosynthesis (left panel) and degradation (right panel) of the *T. foetus* cyst wall. The associated enzyme (EC number) is shown for each step: 3.5.99.6 (Glucosamine-6P isomerase); 2.3.1.4 (Glucosamine 6P N-acetyltransferase); 5.4.2.10 (Phosphoglucosamine mutase); 2.7.7.23 (UDP-N-acetylglucosamine diphosphorylase); 5.1.3.2 (UDP-glucose 4-epimerase); 2.4.1.16 (Chitin synthase); 3.2.1.14 (chitinase); 3.2.1.52 (Beta-N-acetylhexosaminidase); and 2.7.1.59 (N-acetylglucosamine kinase). Green and solid arrows indicate enzymes found in the *T. foetus* genome and are detected in our RNAseq experiment. Red dashed arrows represent enzymes not found in the *T. foetus* genome. Enzymes annotated in the genomes of related protists are also named. NikZ: Nikkomycin Z, UDP-N-acetylglucosamine analogue. Chitin biosynthesis and degradation pathway was adapted from Žárský et al., 2021[65]

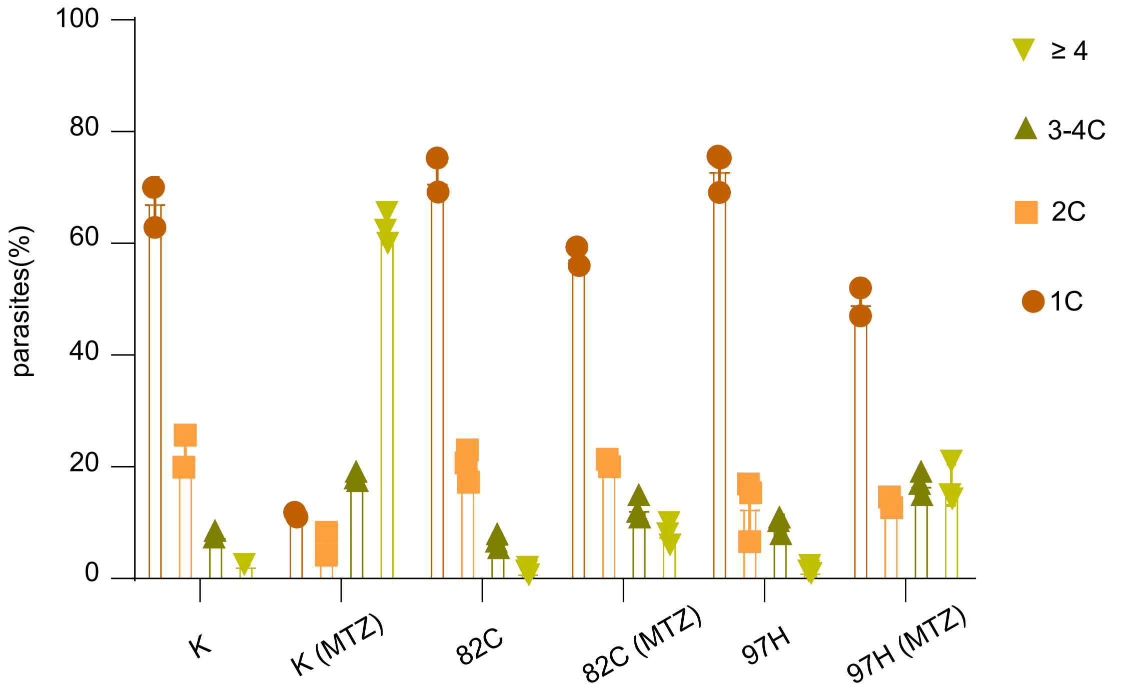

Supplementary Figure 4: DNA content profiles of *T. foetus* strains (K, 82C, and 97H) grown in standard culture medium containing 10 µg/ml metronidazole for 24 h were measured by flow cytometry. In each independent experiment, 10.000 parasites were analyzed. The gating strategy included initial selection of the parasite population based on FSC-SSC parameters followed by singlet discrimination. Each point represents an independent experiment. Data are presented as mean ± s.e.m. (n = 3 independent experiments). Source data are provided as a Source Data file.

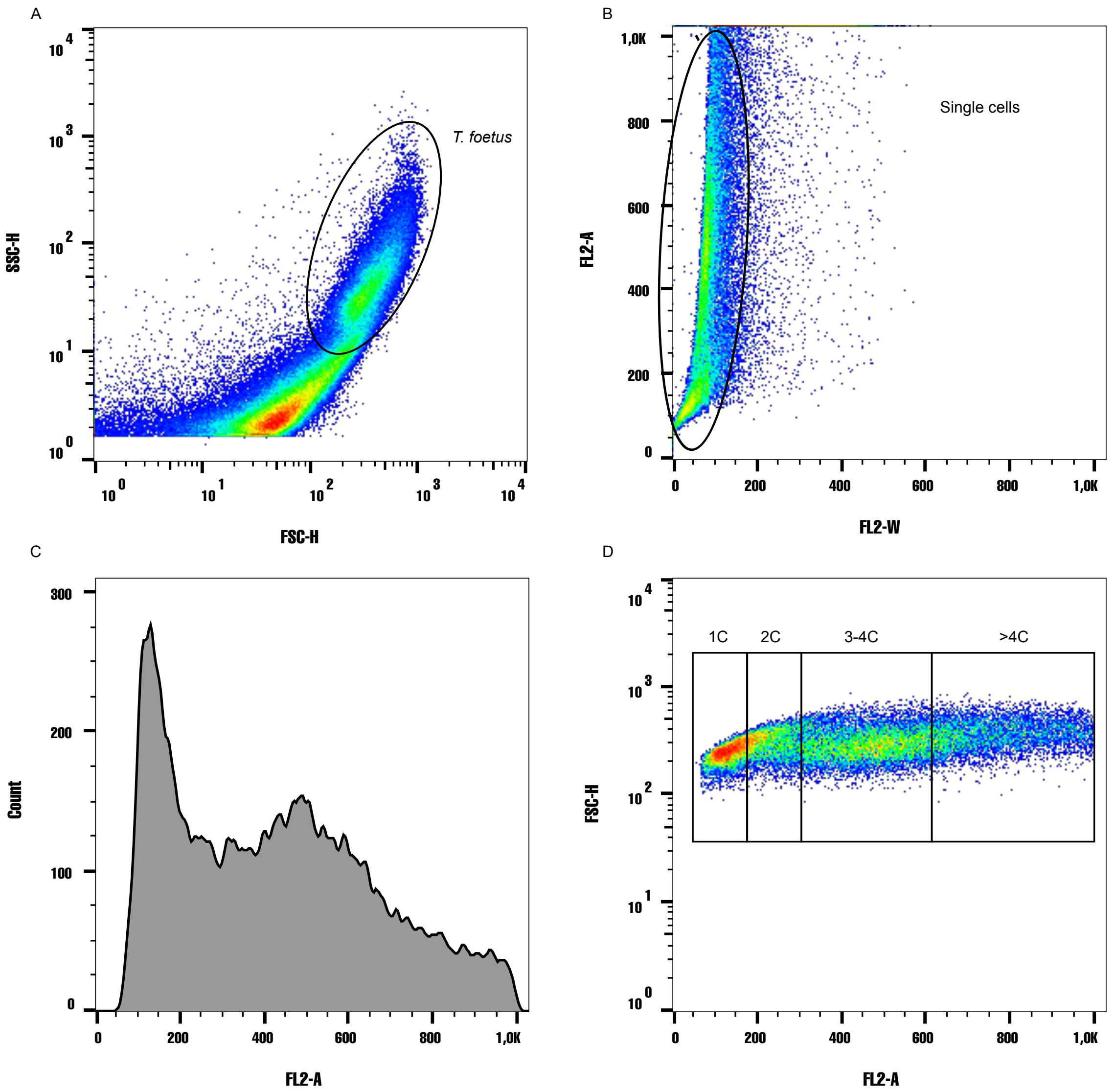

Supplementary Figure 5: Sequential gating strategy for DNA content analysis in *T. foetus*. This gating strategy was used for the analyses presented in Fig. 6d-e of the main manuscript. (A) The main cell population was first selected based on forward and side scatter parameters (FSC-A vs SSC-A) to exclude debris (*T. foetus*). (B) Doublets were excluded by pulse geometry discrimination using FL2-A versus FL2-W, and singlets were selected (Single Cell). (C) Representative histogram of propidium iodide fluorescence intensity (FL2-A) showing DNA content distribution of singlet cells. (D) DNA content (C) regions corresponding to 1C, 2C, 3-4C, and >4C were defined based on the mean fluorescence intensity of each peak ± half of the peak width and subsequently applied on the FSC-A versus FL2-A plot. The same gating boundaries were consistently applied to all experimental conditions. One representative experiment is shown; similar results were obtained in all n = 3 independent biological replicates.

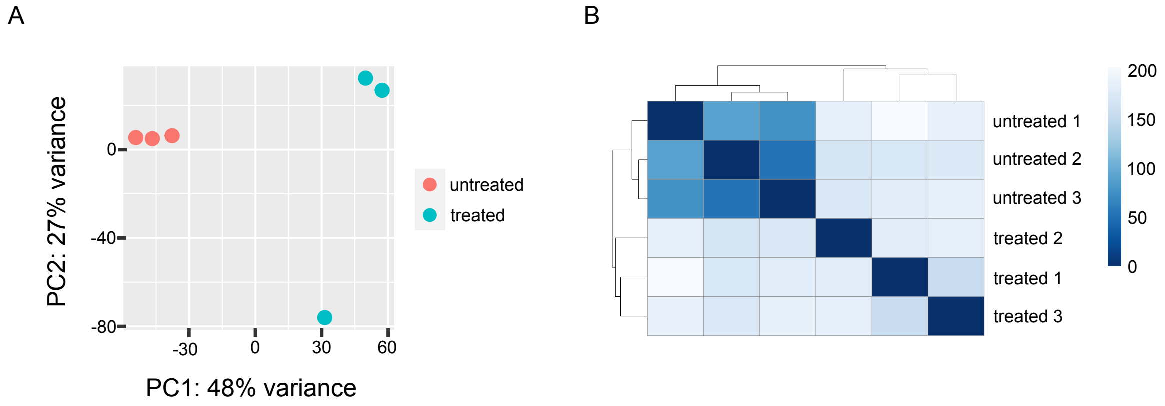

Supplementary Figure 6: Exploratory analysis of RNAseq data for *T. foetus*. Untreated = *T. foetus* K incubated in standard culture media; treated = *T. foetus* K under nutrient restriction conditions for 48 hours. (A) Principal component analysis for treated and untreated *T. foetus* trophozoites RNAseq; (B) A heatmap representation for transformed counts (variance stabilizing transformation). Scale values represent euclidian distance between samples. Source data are provided as a Source Data file.
