# Supplementary material for: True cyst formation underlies persistence and drug tolerance in Tritrichomonas foetus
Source: Nat Commun. 2026 Apr 28;17:5831. doi: 10.1038/s41467-026-71827-9 (PMC13332039; doi:10.1038/s41467-026-71827-9)
Supplement: Supplementary file 2 — Description of Additional Supplementary Files [file 41467_2026_71827_MOESM2_ESM.pdf]

Description of Additional Supplementary Files

Supplementary Data 1: DNA content percentage of different *T. foetus* strains incubated in culture media at pH 8 for 24 and 48 hours. Parasites control: K (c), 82C (c), and 97H (c).

Supplementary Data 2. Summary table of the putative enzymes for *T. foetus* cyst formation. The table is organized as follows: Enzyme (name), Gene ID (from TrichDB.org), Product Description, EC numbers (functional annotation), EC numbers from OrthoMCL (associated by orthology), RNAseq (assembly id), log2 fold change, padj (adjusted p-value), Ctrl1-3 (tpm values for control replicates) and Treatment1-3 (tpm values for treatment replicates).

Supplementary Data 3: Summary table of data and metrics from differential analysis (DESeq2) and GSEA. Table is organized as follows, Assembly Id: assembler id assigned by StringTie software; log2FoldChange: log2 fold change calculated by DESeq2; pvalue: p-value from Wald test; padj: Benjamini-Hochberg adjusted p-values; refGene: reference gene identification number associated with the assembled transcript. Reference identification number were obtained from TrichDB.org (*T. foetus* K1 genome v64); Description: gene product description; Metric: the specific metric used to rank the entire dataset of genes, (log2fc) x (-log10pvalue). P-values were calculated using a two-tailed Wald test and adjusted for multiple testing using the Benjamini-Hochberg method.
