# Supplementary material for: True cyst formation underlies persistence and drug tolerance in Tritrichomonas foetus
Source: Nat Commun. 2026 Apr 28;17:5831. doi: 10.1038/s41467-026-71827-9 (PMC13332039; doi:10.1038/s41467-026-71827-9)
Supplement: Supplementary file 6 — Reporting Summary [file 41467_2026_71827_MOESM6_ESM.pdf]

nature portfolio

Corresponding author(s): VERONICA MABEL COCERES

Last updated by author(s): Mar 30, 2026

Reporting Summary

Nature Portfolio wishes to improve the reproducibility of the work that we publish. This form provides structure for consistency and transparency in reporting. For further information on Nature Portfolio policies, see our Editorial Policies and the Editorial Policy Checklist.

Statistics

For all statistical analyses, confirm that the following items are present in the figure legend, table legend, main text, or Methods section.

n/a	Confirmed	
☐	☒	The exact sample size (*n*) for each experimental group/condition, given as a discrete number and unit of measurement
☐	☒	A statement on whether measurements were taken from distinct samples or whether the same sample was measured repeatedly
☐	☒	The statistical test(s) used AND whether they are one- or two-sided<br>*Only common tests should be described solely by name; describe more complex techniques in the Methods section.*
☐	☒	A description of all covariates tested
☒	☐	A description of any assumptions or corrections, such as tests of normality and adjustment for multiple comparisons
☐	☒	A full description of the statistical parameters including central tendency (e.g. means) or other basic estimates (e.g. regression coefficient) AND variation (e.g. standard deviation) or associated estimates of uncertainty (e.g. confidence intervals)
☐	☒	For null hypothesis testing, the test statistic (e.g. *F*, *t*, *r*) with confidence intervals, effect sizes, degrees of freedom and *P* value noted<br>*Give P values as exact values whenever suitable.*
☒	☐	For Bayesian analysis, information on the choice of priors and Markov chain Monte Carlo settings
☒	☐	For hierarchical and complex designs, identification of the appropriate level for tests and full reporting of outcomes
☒	☐	Estimates of effect sizes (e.g. Cohen's *d*, Pearson's *r*), indicating how they were calculated

Our web collection on statistics for biologists contains articles on many of the points above.

Software and code

Policy information about availability of computer code

Data collection	Confocal images were acquired using FV10-ASW 3.1 software (Olympus). Flow cytometry data were collected using a FACSCalibur flow cytometer (Becton Dickinson). RNA sequencing libraries were prepared and sequenced by Quick Biology using Illumina platforms.
Data analysis	Flow cytometry data were analyzed using FlowJo v7.6. RNA-seq read quality was assessed using FastQC (v0.12.1), and adapter trimming and filtering of low-quality reads were performed using TrimGalore (v0.6.10). Filtered reads were aligned to the reference genome using HISAT2 (v2.2.1), and transcript quantification was performed following the StringTie (v2.1.7) protocol using the prepDE.py (v2.1.7) script to generate the raw count matrix. Differential gene expression analysis and principal component analysis were conducted in R (v4.4.2) using the DESeq2 package (1.48.2). Gene Set Enrichment Analysis was performed using the clusterProfiler package (v4.16.0), and plots were generated using the enrichplot package (v1.28.4). Image processing and analysis were performed using ImageJ 1.45s and Adobe Photoshop.

For manuscripts utilizing custom algorithms or software that are central to the research but not yet described in published literature, software must be made available to editors and reviewers. We strongly encourage code deposition in a community repository (e.g. GitHub). See the Nature Portfolio guidelines for submitting code & software for further information.

Data

Policy information about availability of data

All manuscripts must include a data availability statement. This statement should provide the following information, where applicable:

- Accession codes, unique identifiers, or web links for publicly available datasets
- A description of any restrictions on data availability
- For clinical datasets or third party data, please ensure that the statement adheres to our policy

All data supporting the findings of this study are available within the paper and its Supplementary Information. The RNAseq data generated in this study have been deposited in the GEO database under accession code GSE306990 [https://www.ncbi.nlm.nih.gov/geo/query/acc.cgi?acc=GSE306990].

Research involving human participants, their data, or biological material

Policy information about studies with human participants or human data. See also policy information about sex, gender (identity/presentation), and sexual orientation and race, ethnicity and racism.

Reporting on sex and gender	*Use the terms sex (biological attribute) and gender (shaped by social and cultural circumstances) carefully in order to avoid confusing both terms. Indicate if findings apply to only one sex or gender; describe whether sex and gender were considered in study design; whether sex and/or gender was determined based on self-reporting or assigned and methods used.*<br>*Provide in the source data disaggregated sex and gender data, where this information has been collected, and if consent has been obtained for sharing of individual-level data; provide overall numbers in this Reporting Summary. Please state if this information has not been collected.*<br>*Report sex- and gender-based analyses where performed, justify reasons for lack of sex- and gender-based analysis.*
Reporting on race, ethnicity, or other socially relevant groupings	*Please specify the socially constructed or socially relevant categorization variable(s) used in your manuscript and explain why they were used. Please note that such variables should not be used as proxies for other socially constructed/relevant variables (for example, race or ethnicity should not be used as a proxy for socioeconomic status).*<br>*Provide clear definitions of the relevant terms used, how they were provided (by the participants/respondents, the researchers, or third parties), and the method(s) used to classify people into the different categories (e.g. self-report, census or administrative data, social media data, etc.)*<br>*Please provide details about how you controlled for confounding variables in your analyses.*
Population characteristics	*Describe the covariate-relevant population characteristics of the human research participants (e.g. age, genotypic information, past and current diagnosis and treatment categories). If you filled out the behavioural & social sciences study design questions and have nothing to add here, write "See above."*
Recruitment	*Describe how participants were recruited. Outline any potential self-selection bias or other biases that may be present and how these are likely to impact results.*
Ethics oversight	*Identify the organization(s) that approved the study protocol.*

Note that full information on the approval of the study protocol must also be provided in the manuscript.

Field-specific reporting

Please select the one below that is the best fit for your research. If you are not sure, read the appropriate sections before making your selection.

[x] Life sciences [ ] Behavioural & social sciences [ ] Ecological, evolutionary & environmental sciences

For a reference copy of the document with all sections, see nature.com/documents/nr-reporting-summary-flat.pdf

Life sciences study design

All studies must disclose on these points even when the disclosure is negative.

Sample size	No formal statistical power calculation was performed prior to the study. Sample sizes were determined based on standard practices in in vitro parasitological research and prior experience with similar experimental systems. The use of three independent biological replicates with technical triplicates per condition is widely accepted as sufficient to ensure reproducibility and to allow statistical comparison between groups. This design provides adequate power to detect biologically meaningful differences while maintaining feasibility.
Data exclusions	No data were excluded from the analysis.
Replication	All in vitro experiments were independently replicated three times, and each experimental condition within each experiment was performed in technical triplicate. All replication attempts were successful and yielded consistent results. Multiple strains of Tritrichomonas foetus were included to account for strain-specific variability and to increase the generalizability of the findings.
Randomization	Parasites cryopreserved at different time points were thawed and freshly cultured for each independent experiment to minimize potential culture-adaptation bias. Both the long-maintained laboratory K reference strain and additional strains recently isolated from animals were included to account for potential strain-specific differences and biological variability.For each experiment, parasites were assigned to

experimental groups according to predefined treatment conditions. As this study was conducted in vitro using homogeneous parasite cultures under controlled laboratory conditions, formal randomization was not applicable. All cultures were standardized to the same initial parasite density and maintained under identical environmental conditions prior to treatment to minimize potential confounding effects.

Blinding	Blinding was not applied in this study. Due to the in vitro experimental design, group allocation was defined by controlled laboratory conditions (e.g., treatment vs. control), and outcome measurements (parasite counts, viability assays) were based on objective and standardized criteria. Therefore, blinding was not considered necessary, as the measurements were not subject to subjective interpretation.

Behavioural & social sciences study design

All studies must disclose on these points even when the disclosure is negative.

Study description	*Briefly describe the study type including whether data are quantitative, qualitative, or mixed-methods (e.g. qualitative cross-sectional, quantitative experimental, mixed-methods case study).*
Research sample	*State the research sample (e.g. Harvard university undergraduates, villagers in rural India) and provide relevant demographic information (e.g. age, sex) and indicate whether the sample is representative. Provide a rationale for the study sample chosen. For studies involving existing datasets, please describe the dataset and source.*
Sampling strategy	*Describe the sampling procedure (e.g. random, snowball, stratified, convenience). Describe the statistical methods that were used to predetermine sample size OR if no sample-size calculation was performed, describe how sample sizes were chosen and provide a rationale for why these sample sizes are sufficient. For qualitative data, please indicate whether data saturation was considered, and what criteria were used to decide that no further sampling was needed.*
Data collection	*Provide details about the data collection procedure, including the instruments or devices used to record the data (e.g. pen and paper, computer, eye tracker, video or audio equipment) whether anyone was present besides the participant(s) and the researcher, and whether the researcher was blind to experimental condition and/or the study hypothesis during data collection.*
Timing	*Indicate the start and stop dates of data collection. If there is a gap between collection periods, state the dates for each sample cohort.*
Data exclusions	*If no data were excluded from the analyses, state so OR if data were excluded, provide the exact number of exclusions and the rationale behind them, indicating whether exclusion criteria were pre-established.*
Non-participation	*State how many participants dropped out/declined participation and the reason(s) given OR provide response rate OR state that no participants dropped out/declined participation.*
Randomization	*If participants were not allocated into experimental groups, state so OR describe how participants were allocated to groups, and if allocation was not random, describe how covariates were controlled.*

Ecological, evolutionary & environmental sciences study design

All studies must disclose on these points even when the disclosure is negative.

Study description	*Briefly describe the study. For quantitative data include treatment factors and interactions, design structure (e.g. factorial, nested, hierarchical), nature and number of experimental units and replicates.*
Research sample	*Describe the research sample (e.g. a group of tagged Passer domesticus, all Stenocereus thurberi within Organ Pipe Cactus National Monument), and provide a rationale for the sample choice. When relevant, describe the organism taxa, source, sex, age range and any manipulations. State what population the sample is meant to represent when applicable. For studies involving existing datasets, describe the data and its source.*
Sampling strategy	*Note the sampling procedure. Describe the statistical methods that were used to predetermine sample size OR if no sample-size calculation was performed, describe how sample sizes were chosen and provide a rationale for why these sample sizes are sufficient.*
Data collection	*Describe the data collection procedure, including who recorded the data and how.*
Timing and spatial scale	*Indicate the start and stop dates of data collection, noting the frequency and periodicity of sampling and providing a rationale for these choices. If there is a gap between collection periods, state the dates for each sample cohort. Specify the spatial scale from which the data are taken*
Data exclusions	*If no data were excluded from the analyses, state so OR if data were excluded, describe the exclusions and the rationale behind them, indicating whether exclusion criteria were pre-established.*
Reproducibility	*Describe the measures taken to verify the reproducibility of experimental findings. For each experiment, note whether any attempts to repeat the experiment failed OR state that all attempts to repeat the experiment were successful.*
Randomization	*Describe how samples/organisms/participants were allocated into groups. If allocation was not random, describe how covariates were controlled. If this is not relevant to your study, explain why.*
Blinding	*Describe the extent of blinding used during data acquisition and analysis. If blinding was not possible, describe why OR explain why blinding was not relevant to your study.*

Did the study involve field work? ☐ Yes ☐ No

Field work, collection and transport

Field conditions	*Describe the study conditions for field work, providing relevant parameters (e.g. temperature, rainfall).*
Location	*State the location of the sampling or experiment, providing relevant parameters (e.g. latitude and longitude, elevation, water depth).*
Access & import/export	*Describe the efforts you have made to access habitats and to collect and import/export your samples in a responsible manner and in compliance with local, national and international laws, noting any permits that were obtained (give the name of the issuing authority, the date of issue, and any identifying information).*
Disturbance	*Describe any disturbance caused by the study and how it was minimized.*

Reporting for specific materials, systems and methods

We require information from authors about some types of materials, experimental systems and methods used in many studies. Here, indicate whether each material, system or method listed is relevant to your study. If you are not sure if a list item applies to your research, read the appropriate section before selecting a response.

Materials & experimental systems

n/a	Involved in the study	
☐	☒	Antibodies
☒	☐	Eukaryotic cell lines
☒	☐	Palaeontology and archaeology
☒	☐	Animals and other organisms
☒	☐	Clinical data
☒	☐	Dual use research of concern
☒	☐	Plants

Methods

n/a	Involved in the study	
☒	☐	ChIP-seq
☐	☒	Flow cytometry
☒	☐	MRI-based neuroimaging

Antibodies

Antibodies used	-Anti-TUBA4A (TUBA1) Antibody - Mouse monoclonal, B-5-1-2 (Sigma-Aldrich: T5168).<br><br>-Goat anti-Mouse IgG (H+L) Cross-Adsorbed Secondary Antibody, Alexa Fluor™ 594. Invitrogen/Thermo Fisher Scientific: Catalog # A-11005.
Validation	The primary antibodies used in this study are routinely employed in our laboratory for research on trichomonads (Trichomonas vaginalis, Pentatrichomonas spp., and Tritrichomonas foetus). Specifically, the tubulin marker antibody was recently used to visualize the axostyle, a cytoskeletal structure characteristic of trichomonads, during cell division (https://doi.org/10.1128/spectrum.03251-22).

Plants

Seed stocks	*Report on the source of all seed stocks or other plant material used. If applicable, state the seed stock centre and catalogue number. If plant specimens were collected from the field, describe the collection location, date and sampling procedures.*
Novel plant genotypes	*Describe the methods by which all novel plant genotypes were produced. This includes those generated by transgenic approaches, gene editing, chemical/radiation-based mutagenesis and hybridization. For transgenic lines, describe the transformation method, the number of independent lines analyzed and the generation upon which experiments were performed. For gene-edited lines, describe the editor used, the endogenous sequence targeted for editing, the targeting guide RNA sequence (if applicable) and how the editor was applied.*
Authentication	*Describe any authentication procedures for each seed stock used or novel genotype generated. Describe any experiments used to assess the effect of a mutation and, where applicable, how potential secondary effects (e.g. second site T-DNA insertions, mosiacism, off-target gene editing) were examined.*

Flow Cytometry

Plots

Confirm that:

- [x] The axis labels state the marker and fluorochrome used (e.g. CD4-FITC).
- [x] The axis scales are clearly visible. Include numbers along axes only for bottom left plot of group (a 'group' is an analysis of identical markers).
- [x] All plots are contour plots with outliers or pseudocolor plots.
- [ ] A numerical value for number of cells or percentage (with statistics) is provided.

Methodology

Sample preparation	DNA content was determined by harvesting parasites (5x106) and fixing them in 5 mL of ice-cold 100% ethanol at 4°C overnight. Samples were washed with 1 mL of PBS containing 2% (v/v) horse serum (HS), resuspended in 1 mL of PBS with 180 µg/mL RNase A and 2% (v/v) HS, and incubated at 37°C for 30 min. Parasites were then stained with 25 µg/mL propidium iodide (PI) for 30 min at 37°C prior to flow cytometric analysis.
Instrument	FACSCalibur flow cytometer (BD Biosciences) equipped with a dual-laser system (15 mW, 488 nm argon-ion laser and 635 nm red diode laser)
Software	FlowJo software version 7.6 (Tree Star Inc., Ashland, OR, USA)
Cell population abundance	For each cytometric run, 10,000 events within the gate corresponding to the size and granularity of the parasites were acquired.
Gating strategy	Sequential gating strategy for DNA content analysis in Tritrichomonas foetus.<br>(A) The main cell population was first selected based on forward and side scatter parameters (FSC-A vs SSC-A) to exclude debris (T.foetus).<br>(B) Doublets were excluded by pulse geometry discrimination using FL2-A versus FL2-W, and singlets were selected (Single Cell).<br>(C) Representative histogram of propidium iodide fluorescence intensity (FL2-A) showing DNA content distribution of singlet cells.<br>(D) DNA content (C) regions corresponding to 1C, 2C, 3-4C, and >4C were defined based on the mean fluorescence intensity of each peak ± half of the peak width and subsequently applied on the FSC-A versus FL2-A plot. The same gating boundaries were consistently applied to all experimental conditions. One representative experiment out of n = 3 independent biological replicates is shown.<br><br>For viability analyses, the same initial gate was applied to select parasite events. These were subsequently displayed on an FSC-H versus FL2-A dot plot, where quadrants distinguishing live and dead populations were established using live parasites as the viability control and heat-killed parasites as the death control. The same gating strategy was consistently applied to all remaining samples.

- [x] Tick this box to confirm that a figure exemplifying the gating strategy is provided in the Supplementary Information.
