# Supplementary material for: True cyst formation underlies persistence and drug tolerance in Tritrichomonas foetus
Source: Nat Commun. 2026 Apr 28;17:5831. doi: 10.1038/s41467-026-71827-9 (PMC13332039; doi:10.1038/s41467-026-71827-9)
Supplement: Supplementary file 7 — Transparent Peer Review file [file 41467_2026_71827_MOESM7_ESM.pdf]

nature portfolio

Peer Review File

True cyst formation underlies persistence and drug tolerance in Tritrichomonas foetus

Corresponding Author: Professor Veronica coceres

This file contains all reviewer reports in order by version, followed by all author rebuttals in order by version.

Version 0:

Reviewer comments:

Reviewer #1

(Remarks to the Author)
This report by Iriarte et al. presents exciting new evidence on (pseudo?)cyst formation in the cattle/cat/swine parasite Tritrichomonas foetus. It has been a long-standing matter of debate if trichomonads can form cysts like other (not closely related) parasites such as Giardia lamblia or Entamoeba histolytica.

The authors demonstrate:
a., the formation of a chitinous cell wall
b., the inducibility of encystation under various stress conditions (starvation, pH, metronidazole)
c., a protective effect of the cyst structure against detergents and metronidazole
d., the ability of (pseudo)cysts to excyst upon placement into normal growth medium
e., an increase of DNA content in the cyst-like structures (the corresponding figure didn't make it into the pdf though!)
f., the upregulation in transcription of chitin synthesis-related genes

These are all compelling findings which add to the current status of knowledge. However, the authors aim at establishing the presumptive cyst stage of T. foetus as a clearly defined lifecycle stage. At this point, I fear, a more careful and more detailed approach will be necessary. It remains unclear to the reader in how far the previously described "pseudocysts" differ from the "true cysts" described here. Were "true cysts" ever isolated from bulls or from swine or cats? In a study on Trichomonas vaginalis (Beri et al. 2020), which is also cited but not really discussed in this paper, "cyst-like" structures which are stainable with Calcufluor White were described. These structures are also spherical but somehow lack clearly uniform features. This, in fact, also seems to be the case here. In contrast, cysts from E. histolytica and G. lamblia are stereotypically and very distinctly formed. They have clear morphological features such as multiple nuclei and a distinctive subcellular organization. Of course, the production of a chitinous cell wall constitutes a distinctive feature as compared to the pear-shaped trophozoites, but in how far does it constitute a distinctive feature as compared to "pseudo-cysts"? Importantly, the so-called pseudocyst in Acanthamoeba castellanii (an expression coined by the authors of the cited paper and otherwise not widely used) DO have a cellulose wall. In order to add credibility on this distinction of cysts vs. pseudocysts, a comparative approach between presumptive cysts and presumptive pseudocysts should be performed. Are pseudocysts indeed early developmental stages of the cyst or something different? ARE they different in the first place? The present results should be put into perspective with earlier observations from others, both on T. foetus and also on other trichomonads such as T. vaginalis (e.g. Beri et al., 2020), in a more systematical and discursive manner. The findings should also be put into a wider perspective with regard to the host. Does a true cyst form aid the parasite in its lifecycle? Where can we expect to find it?

Apart from these general considerations some additional experiments would increase the study's impact:
1: the protective effect of the cell wall against metronidazole is intriguing. However, the authors applied concentrations (max. 10 µg/ml) which are lower than serum levels in treated humans and animals. In order to assess if cell wall formation could really be a relevant resistance/evasion mechanism in vivo, a concentration of at least 16 µg/ml should be tested.
2: the authors demonstrate resistance of T. foetus "cysts" against 0.15% sarkosyl. Does the cell wall also provide protection against low pH and desiccation? Both conditions could be of importance for the parasite in its infection cycle.

One minor query:
Lines 401 – 405: There might have been reports on metronidazole resistance in T. foetus but metronidazole and other 5-nitroimidazoles were exclusively barred from use in food-producing animals for safety reasons because a mutagenic effect

of these drugs after consumption cannot be ruled out. Nitroimidazoles are, of course, still in use in veterinary medicine, e.g. against this very parasite: T. foetus infections in cats are routinely treated with ronidazole. Please adjust your wording accordingly!

Reviewer #2

(Remarks to the Author)
In this manuscript, the authors showed that Tritrichomonas foetus, an important parasitic protist of cattle and other animals, is able to form true cysts with a cell wall that contains carbohydrate polymers. This is a very important discovery, which provides multiple implications for epidemiology and transmission of animal trichomoniasis. Thus far, only direct sexual transmission of trophozoites and/or cell wall-free pseudocysts was believed to be involved in transmission between cattle, although the presence of T. foetus in the intestinal tract of cattle, cats, dogs, and pigs is known. The authors demonstrated that cyst formation is induced by nutritional stress, changes in pH, and metronidazole. They also showed that cysts are resistant to relatively high concentrations of metronidazole and thus may represent another mechanism of metronidazole resistance. The formation of cysts is supported by fluorescence and electron microscopy (SEM, and TEM), which showed the morphology of the cyst wall, significant changes in the cytoskeleton, and cytoplasmic content. Overall, this is a very strong contribution, with compelling multiple complementary lines of evidence, concerning true cyst formation. It will represent an important contribution to the understanding of the parasite biology and transmission. However, I also have a few issues that need to be considered.
Major
1. The major unanswered question is the nature of the cell wall polymer. Although the direct molecular analysis is beyond the scope of this paper, the authors can draw the metabolic pathways for the synthesis and degradation of known polymers in cell walls of other protists (cellulose, chitin, GalNAc), and based on their transcriptomic data and genome sequence, clarify which components of these pathways are present in T. foetus. Which enzymes required for N-acetyl-D-glucosamine are actually identified in T. foetus? This will help with the discussion – lines 338-346. In the current form, the opinion of the authors on this key issue is not clear. They are referring to a paper Kneipp et al., 1998 that detected chitin by anti-chitin antibody. However, Kneipp et al observed putative chitin in trophozoits, not in cysts. The authors should clarify whether T. foetus possesses enzymes such as chitin synthase and chitinase. If not, chitin is possibly not present. See, for example, chitin synthesis in Mastigamoeba balamuthi (Zarsky et al., 2021).
2. Line 189 "…the formation of cyst-like structures in multiple T. foetus strains, accompanied by increased DNA content and the presence of multinucleated forms".
In giardia and other protists, the true cysts usually have a defined number of nuclei. In Figure 6F there are multiple nuclei visible. Please consider, in addition to cytometry, the calculation of nuclei in cysts. What is the variability?
3. Line 230 Metronidazole treatment. The ability of T. foetus to survive for 24-48 hours up to 10 ug/ml of metronidazole is very surprising. In previous studies, susceptibility of T. foetus to metronidazole was tested using incubation of trophozoites with various concentrations of metronidazole, and after 48 the MLC (minimal lethal concentration) was determined by viability test using re-cultivation of metronidazole-treated trichomonads in drug-free medium as performed in this study. T. foetus was usually not able to survive 2.5 ug/ml (Kulda et al., 1984). Please comment on this significant difference.
4. Line 385 "… while excystation is triggered when the environment shifts to pH 6.4, which mimics the vaginal mucus of cows."
Please provide a reference. It has been reported that in vivo the pH of bovine vaginal epithelium is approximately 7.6 (Corbeil et al. 1989). Optimal growth is at 6.6 to 7.8, and the TYM media for T. foetus is usually adjusted to 7.5. Please clarify these issues with optimal pH.
5. Line 130-135. "Additionally, we observed redistribution of cytoplasmic content in cysts by fluorescence microscopy."
Hydrogenosomes in Figure 4A have very unusual morphology and localization. Authors should comment on this, and it would be beneficial to support this observation by electron microscopy. It is not necessary to perform immunoelectron microscopy; hydrogensomes have a characteristic structure.

Minor
Line 60 Trichomitus batachorum, typo - batrachorum
Line 68, 104, 337, 405. Please do not repeat at multiple sites "… for the first time…"
Line 173 Sarkosyl resistance assays showed that the K strain formed 4.17% and 0.75% cysts at 24 and 48 hours, respectively; the 82C strain produced 1.08% (24h) and 3% (48h); and the 97H strain formed 0.42% (24h) and 0.67%
Please comment on why there is a decrease in time in the case of K strain and an opposite trend in the other two strains?
Line 149 "At 96 hours, the rates increased to 5.5% for K, 5.83% for C, and 0.566% for 97H (Fig. 5A)." Please, modify the sentence; in the case of 97H, there is no increase.
Figure 2c-d: Scale bar is missing
Figure 5 (B) and (C) are not indicated in the Figure
Figure 6 B-E are not indicated in the Figure
Fig 8 The Enrichment plot needs a clear description of the information embedded in the three parts of Fig 8A.
Fig 8 and Fig 9, in the legend, please indicate the related supplementary table with the corresponding data analysis.
Line 415. Include information about the origin of strains 82C and 97H.

Version 1:

Reviewer comments:

Reviewer #1

(Remarks to the Author)
The authors have undertaken a great effort to improve the manuscript according to my queries and suggestions, including several series of novel experiments. All of these further corroborate the authors' claims. I therefore have no further objections to the publication of this important work.

Reviewer #2

(Remarks to the Author)
The authors have carefully addressed most of the questions raised in the review. I have only a minor issue remaining, which should still be considered before final acceptance.
The structures labeled with the anti-Hmp24 antibody in Figure 3 do not match the usual hydrogenosomal morphology, casting doubt on their identification as hydrogenosomes (as I noted previously). To resolve this, the authors should either use at least two antibodies for co-localization within the organelle or provide electron microscopy support, per my prior suggestion. Notably, no reference is provided for the source of the anti-Hmp24 antibody.
Nevertheless, I appreciate the authors' clarification that Figure 3 primarily focuses on the cytoskeleton/tubulin. A straightforward solution would be to remove the Hmp24 signal from Figure 3 (along with related comments) and address hydrogenosome morphology in cysts in a future study.

Open Access This Peer Review File is licensed under a Creative Commons Attribution 4.0 International License, which permits use, sharing, adaptation, distribution and reproduction in any medium or format, as long as you give appropriate credit to the original author(s) and the source, provide a link to the Creative Commons license, and indicate if changes were made.
In cases where reviewers are anonymous, credit should be given to 'Anonymous Referee' and the source.
The images or other third party material in this Peer Review File are included in the article's Creative Commons license, unless indicated otherwise in a credit line to the material. If material is not included in the article's Creative Commons license and your intended use is not permitted by statutory regulation or exceeds the permitted use, you will need to obtain permission directly from the copyright holder.
To view a copy of this license, visit https://creativecommons.org/licenses/by/4.0/

Initially, we would like to thank the Reviewers and the Editor for their valuable time and comments, which have undoubtedly contributed to improving the manuscript. We greatly appreciate their suggestions and critical observations regarding the quality, strengths, and limitations of our main findings and writing. We are also grateful for the opportunity to address the questions and concerns raised about our manuscript.

We have carefully considered all the points raised by the referees, and the corresponding revisions are highlighted in blue in the revised version of the manuscript.
Thank you for your efforts on our behalf, and we look forward to hearing from you at your earliest convenience.

Reviewer #1:

This report by Iriarte et al. presents exciting new evidence on (pseudo?) cyst formation in the cattle/cat/swine parasite *Tritrichomonas foetus*. It has been a long-standing matter of debate if trichomonads can form cysts like other (not closely related) parasites such as *Giardia lamblia* or *Entamoeba histolytica*.

The authors demonstrate:
a., the formation of a chitinous cell wall
b., the inducibility of encystation under various stress conditions (starvation, pH, metronidazole)
c., a protective effect of the cyst structure against detergents and metronidazole
d., the ability of (pseudo)cysts to excyst upon placement into normal growth medium
e., an increase of DNA content in the cyst-like structures (the corresponding figure didn't make it into the pdf though!)

Response: We thank the reviewer for this observation. In the original version of the manuscript, panel labels D and E were missing from Figure 6. This labeling error has now been corrected, and the figure has been updated accordingly. **No new data or analyses were added.**

f., the upregulation in transcription of chitin synthesis-related genes

These are all compelling findings which add to the current status of knowledge. However, the authors aim at establishing the presumptive cyst stage of *T. foetus* as a clearly defined lifecycle stage. At this point, I fear, a more careful and more detailed approach will be necessary. It remains unclear to the reader in how far the previously described "pseudocysts" differ from the "true cysts" described here. Were "true cysts" ever isolated from bulls or from swine or cats? In a study on Trichomonas vaginalis (Beri et al. 2020), which is also cited but not really discussed in this paper, "cyst-like" structures which are stainable with Calcofluor White were described. These structures are also spherical but somehow lack clearly uniform features. This, in fact, also seems to be the case here. In contrast, cysts from *E. histolytica* and *G. lamblia* are stereotypically and very distinctly

formed. They have clear morphological features such as multiple nuclei and a distinctive subcellular organization. Of course, the production of a chitinous cell wall constitutes a distinctive feature as compared to the pear-shaped trophozoites, but in how far does it constitute a distinctive feature as compared to "pseudo-cysts"? Importantly, the so-called pseudocyst in *Acanthamoeba castellanii* (an expression coined by the authors of the cited paper and otherwise not widely used) DO have a cellulose wall. In order to add credibility on this distinction of cysts vs. pseudocysts, a comparative approach between presumptive cysts and presumptive pseudocysts should be performed. Are pseudocysts indeed early developmental stages of the cyst or something different? ARE they different in the first place? The present results should be put into perspective with earlier observations from others, both on *T. foetus* and also on other trichomonads such as *T. vaginalis* (e.g. Beri et al., 2020), in a more systematical and discursive manner. The findings should also be put into a wider perspective with regard to the host. Does a true cyst form aid the parasite in its lifecycle? Where can we expect to find it?

Response: We thank the reviewer for this thoughtful and constructive comment. Firstly, we have substantially expanded and revised the **Discussion section (page 9, lines 407–410)** to better contextualize our findings within the existing literature on *T. vaginalis* (Beri et al., 2020). Below, we address the specific points raised:

(A) Isolation of true cysts from hosts

-Type of response: clarification (no new data).

To date, true cyst structures of *T. foetus* have not been conclusively isolated from infected hosts (bulls, swine, or cats). Previous studies have reported pseudocyst-like or cyst-like forms in fresh preputial secretion samples from bulls[1] and in fecal samples from cats with chronic diarrhea[2]; however, these structures were not completely characterized and therefore could not be conclusively classified as true cysts. Related to this the aim of this study was rather to establish experimentally supported criteria to identify and characterize stress-induced structures under controlled conditions, providing a framework for future *in vivo* studies.

(B) Distinction between pseudocysts and true cysts

-Type of response: clarification.

In the bibliography, true cysts are defined as structures enclosed by a cyst wall composed of polysaccharides (typically chitin or chitin-like polymers) and associated proteins. This specialized wall confers resistance to desiccation, pH and osmotic stress, temperature fluctuations, and chemical agents. Cyst formation is accompanied by coordinated morphological, biochemical, and transcriptomic changes, and mature cysts retain the capacity to excyst when environmental conditions become favorable. In contrast, pseudocysts are described as transient and reversible forms produced under stress

conditions, which lack a newly synthesized cyst wall, remain bounded only by the plasma membrane, and rapidly revert to trophozoites once the stress is removed.

In our view, previous reports did not fully characterize these structures in *T. vaginalis* and *T. foetus* and therefore referred to them conservatively as pseudocyst-like or cyst-like forms. In this study, to confirm the existence of true cysts in *T. foetus*, we examined multiple stress conditions during the encystation process, including both severe and moderate stresses in different *T. foetus* strains, we evaluated the excystation process, the presence of a fibrillar cyst wall was demonstrated by transmission electron microscopy (TEM), we investigated the cyst wall formation pathway through transcriptomic analysis, and we proposed cyst wall biosynthesis and degradation pathway comparing *T. foetus* cysts with those described in other protists. We also demonstrated that cyst wall-enriched preparations are composed predominantly of amino sugars and neutral hexoses. The high levels of glucosamine and galactosamine are consistent with polymers of N-acetylated hexosamines, supporting the presence of a rigid, polysaccharide-rich cyst wall. These biochemical findings independently corroborate the microscopy-based detection of β-linked glycans and align with the transcriptomic enrichment of carbohydrate biosynthesis pathways observed in cysts formed under stress conditions.

Finally, we validated the functional relevance of the *T. foetus* cyst wall biosynthetic pathway by pharmacological inhibition using nikkomycin Z, a competitive analogue of UDP-N-acetylglucosamine and an inhibitor of chitin synthesis. Co-treatment of *T. foetus* trophozoites with nikkomycin Z markedly increased parasite susceptibility to metronidazole, demonstrating that cyst wall formation plays a key role as a protective barrier and contributes to drug resistance in this protozoan.

Collectively, these aspects correspond to criteria that are commonly considered when assessing the existence of bona fide cyst formation in other protozoans.

(C) Evidence for a cyst maturation process

-Type of response: clarification and additional experiments.

Based on our experimental observations, we propose that pseudocyst-like forms may represent an intermediate stage during cyst maturation. To support this interpretation, we applied for a combined CFW and Evans Blue staining protocol at different time points during the encystation process of *T. foetus*, following an approach originally described for Entamoeba encystation[3]:

Early-stage parasites showed strong Evans Blue uptake together with diffuse CFW staining (CFW^{+}/EB^{+}), whereas intermediate forms partially excluded Evans Blue and displayed a more defined CFW-positive wall. Fully developed structures completely excluded Evans Blue, exhibited intense CFW staining, and were resistant to sarkosyl treatment (CFW^{+}/EB^{-}),

consistent with the presence of a mature cyst wall. Accordingly, we operationally defined mature cysts as CFW-positive and detergent-resistant structures, whereas immature forms were defined as those unable to withstand detergent treatment. Based on these observations, we refined the descriptions of these structures in the manuscript and added **Supplementary Figure 1,** and the corresponding text has been revised accordingly **(section Results page 3, lines 110-118)**.

Related to this, we highlight that in previous reports, pseudocyst formation in *T. foetus* was induced for 4 hours at 4 °C, and for reversibility analysis, the samples were returned to 37 °C for 4 hours. We consider that these short induction times, and particularly the rapid reversion observed, are consistent with the formation of pseudocysts rather than true cysts, although complementary studies are needed to confirm this observation[5-6].

Finally, the potential relevance of a cyst stage for parasite persistence, environmental survival, and transmission of *T. foetus* remains an important question for future investigations.

Were "true cysts" ever isolated from bulls or from swine or cats? In a study on *Trichomonas vaginalis* (Beri et al. 2020), which is also cited but not really discussed in this paper, "cyst-like" structures which are stainable with Calcofluor White were described. These structures are also spherical but somehow lack clearly uniform features.

-Type of response: clarification.

Although *T. foetus* has been detected in these hosts (bulls, swine and cats) the morphological stages described in the literature correspond either to trophozoites or to stress-induced, pseudocyst-like rounded forms rather than to fully differentiated cysts sensu stricto. Whether fully differentiated cysts occur *in vivo* and contribute to parasite transmission therefore remains an open question that warrants further investigation. In this context, the formation of cyst-like rounded structures has recently been observed in *T. foetus* parasites incubated in water or in bovine fecal extracts[4].

In particular, the study by Beri et al. (2020) in *T. vaginalis* described spherical structures that stained with Calcofluor White (CFW) but lacked uniform morphological features. In our view, the CFW-stained structures shown in Figures 3B and 6 do not appear as clearly defined, uniform forms. We consider that the use of CFW as unique staining technique for identification of cyst structures makes it difficult to distinguish pseudocysts from true cyst-like structures, and these structures could therefore represent either pseudocysts or cysts in maturation process. We consider the work of Beri et al. to be highly valuable; however, the authors did not definitively characterize these structures. Transmission electron microscopy did not clearly reveal the cyst wall architecture (Figure 8), nor was there any analysis of the genes involved in its biosynthetic pathway or a detailed characterization of the wall's sugar composition. Furthermore, the authors used sterile double-distilled water

to enrich cyst-like structures through osmotic stress by eliminating trophozoites; in our experience, however, detergent treatment yields more consistent and reproducible results. Therefore, it is possible that, in some analyses, intermediate forms were not completely removed, which could also account for the different morphologies observed as CFW-positive.

The present results should be put into perspective with earlier observations from others, both on *T. foetus* and also on other trichomonads such as *T. vaginalis* (e.g. Beri et al., 2020), in a more systematical and discursive manner. The findings should also be put into a wider perspective with regard to the host. Does a true cyst form aid the parasite in its lifecycle? Where can we expect to find it?

-Type of response: clarification.

With regard to the reviewer's question concerning the potential role of cyst formation in the parasite life cycle and its possible in vivo localization, previous studies have shown that *T. foetus* is able to survive passage through the gastrointestinal tract and subsequently reach the urogenital tract via fecal contamination during defecation (descending route). In addition, we have shown that *T. foetus* can persist for several days in feces and remain viable in non-distilled water for up to 72 hours[4].

While these observations support the notion that environmental persistence may play an important role in parasite transmission, the specific contribution of fully differentiated cysts to this process has not yet been demonstrated *in vivo*. To date, the morphological stages detected in fecal samples have not been fully characterized and therefore cannot be conclusively defined as true cysts. Nevertheless, these findings raise the possibility that cyst formation could facilitate parasite survival outside the host, particularly in feces and water-contaminated environments, thereby contributing to transmission. Whether fully differentiated cysts occur in vivo and directly aid *T. foetus* transmission therefore remains an open question that warrants further investigation.

Apart from these general considerations, some additional experiments would increase the study's impact:

1: the protective effect of the cell wall against metronidazole is intriguing. However, the authors applied concentrations (max. 10 µg/ml) which are lower than serum levels in treated humans and animals. In order to assess if cell wall formation could really be a relevant resistance/evasion mechanism in vivo, a concentration of at least 16 µg/ml should be tested.

-Type of response: additional experiments.

We thank the reviewer for this insightful comment. In response, we have performed additional experiments testing metronidazole at 16 µg/ml and 32 µg/ml. This data have been incorporated into the manuscript (see **Section Results, pages 7-8, lines 307-326)**.

2: the authors demonstrate resistance of *T. foetus* “cysts” against 0.15% sarkosyl. Does the cell wall also provide protection against low pH and desiccation? Both conditions could be of importance for the parasite in its infection cycle.

-Type of response: additional experiments.

We thank the reviewer for this valuable observation. In response, we evaluated the resistance of *T. foetus* cyst-like structures to low pH and desiccation. Our results indicate that the cyst wall provides partial protection under both acidic and desiccating conditions during in vitro assays. These findings have been incorporated into the manuscript **(Section Results, pages 5-6, lines 224-233)**.

One minor query:

Lines 401 – 405: There might have been reports on metronidazole resistance in *T. foetus* but metronidazole and other 5-nitroimidazoles were exclusively barred from use in food-producing animals for safety reasons because a mutagenic effect of these drugs after consumption cannot be ruled out. Nitroimidazoles are, of course, still in use in veterinary medicine, e.g. against this very parasite: *T. foetus* infections in cats are routinely treated with ronidazole. Please adjust your wording accordingly!

Response: Thanks for the observation; we modified the manuscript.

References

1. Pereira-Neves A, Campero C.M, Martínez A, Benchimol M. Identification of *Tritrichomonas foetus* pseudocysts in fresh preputial secretion samples from bulls. Vet Parasitol. 2011 Jan 10;175(1-2):1-8. doi: 10.1016/j.vetpar.2010.10.007. Epub 2010 Oct 14.

2. Zanzani SA, Gazzonis AL, Scarpa P, Olivieri E, Balzer H, Manfredi MT. Coinfection with *Tritrichomonas foetus* and *Giardia duodenalis* in Two Cats with Chronic Diarrhea. Case Rep Vet Med. 2016 Sep 6:2016:5705168. doi: 10.1155/2016/5705168. eCollection 2016.

3. Mi-ichi F, Miyake Y, Tam VK, Yoshida H. A flow cytometry method for dissecting the cell differentiation process of *Entamoeba* encystation. Front Cell Infect Microbiol. 2018; 8:250. doi:10.3389/fcimb.2018.00250.

4. Martínez CI, Iriarte LS, Salas N, Alonso AM, Pruzzo CI, dos Santos Melo T, Pereira-Neves A, de Miguel N, Coceres VM. Prolonged survival of venereal *Tritrichomonas foetus* parasite in the gastrointestinal tract, bovine fecal extract, and water. Microbiol Spectr. 2023;11(6):e00429-23. doi:10.1128/spectrum.00429-23.

5. Pereira-Neves A, Benchimol M. *Tritrichomonas foetus*: budding from multinucleated pseudocysts. *Protist.* 2009;160(4):536–51. doi:10.1016/j.protis.2009.05.001.

6. de Andrade Rosa I, de Souza W, Benchimol M. Changes in the structural organization of the cytoskeleton of *Tritrichomonas foetus* during trophozoite-pseudocyst transformation. *Micron.* 2015;73:28-35. doi:10.1016/j.micron.2015.03.008.

Reviewer #2:

In this manuscript, the authors showed that *Tritrichomonas foetus*, an important parasitic protist of cattle and other animals, is able to form true cysts with a cell wall that contains carbohydrate polymers. This is a very important discovery, which provides multiple implications for epidemiology and transmission of animal trichomoniasis. Thus far, only direct sexual transmission of trophozoites and/or cell wall-free pseudocysts was believed to be involved in transmission between cattle, although the presence of *T. foetus* in the intestinal tract of cattle, cats, dogs, and pigs is known. The authors demonstrated that cyst formation is induced by nutritional stress, changes in pH, and metronidazole. They also showed that cysts are resistant to relatively high concentrations of metronidazole and thus may represent another mechanism of metronidazole resistance. The formation of cysts is supported by fluorescence and electron microscopy (SEM, and TEM), which showed the morphology of the cyst wall, significant changes in the cytoskeleton, and cytoplasmic content. Overall, this is a very strong contribution, with compelling multiple complementary lines of evidence, concerning true cyst formation. It will represent an important contribution to the understanding of the parasite biology and transmission. However, I also have a few issues that need to be considered.

Major

1. The major unanswered question is the nature of the cell wall polymer. Although the direct molecular analysis is beyond the scope of this paper, the authors can draw the metabolic pathways for the synthesis and degradation of known polymers in cell walls of other protists (cellulose, chitin, GalNAc), and based on their transcriptomic data and genome sequence, clarify which components of these pathways are present in *T. foetus*. Which enzymes required for N-acetyl-D-glucosamine are actually identified in *T. foetus*? This will help with the discussion – lines 338-346. In the current form, the opinion of the authors on this key issue is not clear. They are referring to a paper Kneipp et al., 1998 that detected chitin by anti-chitin antibody. However, Kneipp et al observed putative chitin in trophozoits, not in cysts. The authors should clarify whether *T. foetus* possesses enzymes such as chitin synthase and chitinase. If not, chitin is possibly not present. See, for example, chitin synthesis in *Mastigamoeba balamuthi* (Zarsky et al., 2021).

-Type of response: new analyses and additional experiments.

We thank the reviewer for this valuable observation. In the revised version of the manuscript, we have expanded the discussion of the putative cyst wall composition by incorporating a comparative analysis of metabolic pathways involved in the synthesis and degradation of major cell wall polymers described in other protists. **(Section Results, pages 6, lines 262-272; Supplementary Figure 3 and Supplementary Table I)**.

In addition, we incorporated an analysis of the monosaccharide composition of cyst wall–enriched preparations, which was carried out through acid hydrolysis followed by HPAEC-PAD **(Section Results, pages 6-7, lines 274-299 and Table II)**.

2. Line 189 "…the formation of cyst-like structures in multiple *T. foetus* strains, accompanied by increased DNA content and the presence of multinucleated forms". In giardia and other protists, the true cysts usually have a defined number of nuclei. In Figure 6F there are multiple nuclei visible. Please consider, in addition to cytometry, the calculation of nuclei in cysts. What is the variability?

-Type of response: additional experiments.

We appreciate the reviewer's suggestion and have incorporated the requested information into the revised version of the manuscript **(Section Results, page 5, lines 202-209 and Supplementary Figure 2)**.

3. Line 230 Metronidazole treatment. The ability of *T. foetus* to survive for 24-48 hours up to 10 ug/ml of metronidazole is very surprising. In previous studies, susceptibility of *T. foetus* to metronidazole was tested using incubation of trophozoites with various concentrations of metronidazole, and after 48 the MLC (minimal lethal concentration) was determined by viability test using re-cultivation of metronidazole-treated trichomonads in drug-free medium as performed in this study. *T. foetus* was usually not able to survive 2.5 ug/ml (Kulda et al., 1984). Please comment on this significant difference.

-Type of response: clarification

Based on our experience, we consider that the differences observed could be attributable to several factors. For example, in the context of our *in vitro* studies, we observed variability in excystation efficiency, which appeared to depend on the type of stress applied and, to some extent, on the strain analyzed. In particular, metronidazole treatments showed considerable heterogeneity. Following a 24 hour exposure, excystation of strain K was detected at 24 h, whereas strains 97H and 82C exhibited excystation at later time points (72 h and 120 h, respectively). After 48 hours of metronidazole treatment, excystation was observed at 48 h only in strains K and 82C. Moreover, we have observed that several experimental factors may influence the excystation process *in vitro*: A) Under certain stress conditions, prolonged maintenance of cysts in culture appeared to require renewal of the culture medium to facilitate reversion. B) Variability in culture medium composition, including different commercial sources of yeast extract or maltose, may affect excystation efficiency. C) Parasites maintained in *in vitro* culture for extended periods tended to exhibit reduced excystation efficiency. Performing the assays with recently thawed parasites is preferable.

Regarding the study by Kulda et al. (1984), we consider that certain methodological differences, such as the use of a single strain, the absence of alternative viability

assessments on parasite pellets, and the lack of culture medium renewal, may have contributed to the differences observed. However, additional experiments would be required to directly test these possibilities.

4. Line 385 "… while excystation is triggered when the environment shifts to pH 6.4, which mimics the vaginal mucus of cows."
Please provide a reference. It has been reported that in vivo the pH of bovine vaginal epithelium is approximately 7.6 (Corbeil et al. 1989). Optimal growth is at 6.6 to 7.8, and the TYM media for *T. foetus* is usually adjusted to 7.5. Please clarify these issues with optimal pH.

-Type of response: clarification

This is an important point raised by the reviewer, and we truly appreciate it. We noted across multiple reports that pH measurements of the bovine reproductive tract vary considerably among studies and experimental conditions. Several articles describe pH differences across the anatomical regions of the reproductive tract (vagina, cervix, and uterus), as well as across the different stages of the estrous cycle of cows. Moreover, it has been demonstrated that diet can also influence reproductive tract pH. For example, some authors have reported a vaginal pH range of 5.52 to 8.60. In a study of 400 cows, the vaginal pH near the cervix ranged from 5.52 to 8.00, with nearly 50% of the values falling between 6.51 and 7.00, and 93.5% between 6.01 and 7.50[1]. Similarly, in another study involving 54 cows examined at different stages of the estrous cycle, a decrease in vaginal pH (increased acidity) was observed during estrus[2], indicating that reproductive stage also influences vaginal acidity. In addition, a decrease in uterine pH has been reported during the luteal phase in cows fed diets high in rumen-degradable protein[3], further emphasizing that both physiological and nutritional factors can modulate pH within the reproductive tract. Furthermore, excessive dietary protein has been associated with reduced uterine pH and decreased fertility, negatively affecting embryo implantation and development[4–5]. In this context, considering that pH decreases can impair embryo implantation, and that *T. foetus* infection is known to cause early embryonic losses, we believe it is essential to further investigate the relationship between *T. foetus* and pH fluctuations within the reproductive tract. Our research group is currently evaluating this potential association in cows.

Additionally, based on our experience related to in vitro cultures, we routinely maintain *T. foetus* in TYM at pH 6.5, under which conditions the parasites exhibit normal growth. We have observed reduced growth rates when parasites are incubated at lower pH values (e.g., pH 5).

5. Line 130-135. "Additionally, we observed redistribution of cytoplasmic content in cysts by fluorescence microscopy."
Hydrogenosomes in Figure 4A have very unusual morphology and localization. Authors should comment on this, and it would be beneficial to support this observation by

electron microscopy. It is not necessary to perform immunoelectron microscopy; hydrogensomes have a characteristic structure.

-Type of response: clarification

We appreciated the reviewer's comment. We agree with the reviewer that the image is not ideal for illustrating hydrogenosomes. We selected it because it was the most representative image for demonstrating the presence or absence of tubulin, which was the primary focus of that figure. In the following figure, we show images of hydrogenosomes localization in mono- and multinucleated cysts during the early stages of their formation.

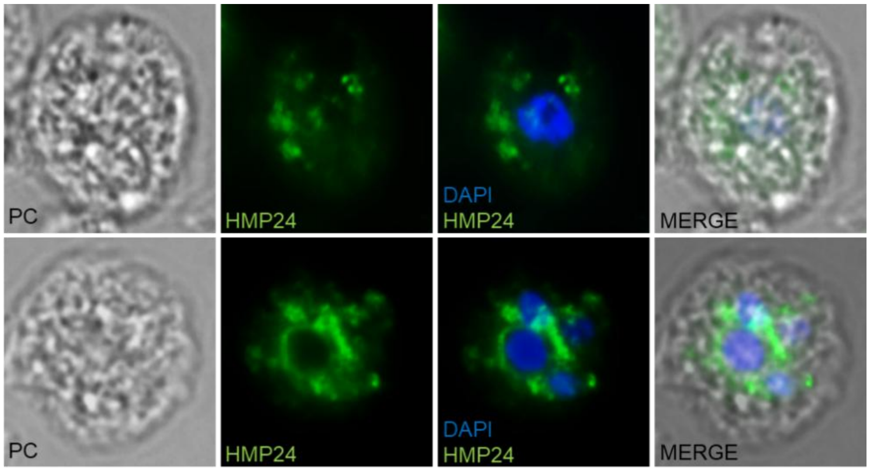

Minor:

-Line 60 *Trichomitus batachorum*, typo - *batrachorum*

Line 68, 104, 337, 405. Please do not repeat at multiple sites "… for the first time…"

Response: Thanks, we modified the sentences.

-Line 173 Sarkosyl resistance assays showed that the K strain formed 4.17% and 0.75% cysts at 24 and 48 hours, respectively; the 82C strain produced 1.08% (24h) and 3% (48h); and the 97H strain formed 0.42% (24h) and 0.67%

Please comment on why there is a decrease in time in the case of K strain and an opposite trend in the other two strains?

Response: We thank the reviewer for this observation. We consider that the decrease observed over time in strain K could be related to the fact that this strain has been maintained in research laboratories for several years, which could have affected its encystation dynamics.

-Line 149 "At 96 hours, the rates increased to 5.5% for K, 5.83% for C, and 0.566% for 97H (Fig. 5A). "Please, modify the sentence; in the case of 97H, there is no increase.

Response: Thanks; we modified the sentence.

-Figure 2c-d: Scale bar is missing

Response: We have now included the scale bar in the figures 2C-D.

-Figure 5 (B) and (C) are not indicated in the Figure

Response: Thanks for the observation; we have corrected the mistake.

-Figure 6 B-E are not indicated in the Figure

Response: We have corrected the mistake.

-Fig 8 The Enrichment plot needs a clear description of the information embedded in the three parts of Fig 8A.

Response: We thank the reviewer for this comment and have included a detailed description of the information represented in the three parts of Figure 8A in the revised manuscript **(page 27, lines 1050-1058)**.

-Fig 8 and Fig 9, in the legend, please indicate the related supplementary table with the corresponding data analysis.

Response: We thank the reviewer for this suggestion and have updated the legends of Figures 8 and 9 to indicate the corresponding supplementary tables containing the related data analyses **(page 28, lines 1074-1082)**.

-Line 415. Include information about the origin of strains 82C and 97H.

Response: Thanks, we have included this information in the manuscript **(Section Materials and Methods, lines 526-529)**.

References

1. Dougherty, R. W.: In vivo determination of the hydrogen ion concentration of the vaginas of dairy cows. North Am. Vet. 22:216-219, 1941.

2. Schilling, E. and Zust, J.: Diagnosis of oestrus and ovulation in cows by pH-measurements intra vagina and by apparent viscosity of vaginal mucus. J. Reprod. Fert. 15:307-311, 1968. doi:10.1530/jrf.0.0150307

3. Butler, W.R.: Effect of Protein Nutrition on Ovarian and Uterine Physiology in Dairy Cattle. Journal of Dairy Science 81(9):2533-9. October 1998. doi:10.3168/jds.S0022-0302(98)70146-8

4. Ibtisham F, et al. Effect of nutrition on reproductive efficiency of dairy animals. Med. Weter. 2018;74(6):356–61. doi:10.21521/mw.6025.

5. Salo S. Effects of quality and amounts of dietary protein on dairy cattle reproduction and the environment. J Dairy Vet Sci. 2018;5:1–7. doi:10.19080/JDVS.2018.05.555675.

We thank the reviewers once again for their valuable comments and suggestions.
We have considered the points raised by the referees, and the corresponding revisions are highlighted in blue in the revised version of the manuscript.

Reviewer #1:

The authors have undertaken a great effort to improve the manuscript according to my queries and suggestions, including several series of novel experiments. All of these further corroborate the authors' claims. I therefore have no further objections to the publication of this important work.

Reviewer #2:

The authors have carefully addressed most of the questions raised in the review. I have only a minor issue remaining, which should still be considered before final acceptance. The structures labeled with the anti-Hmp24 antibody in Figure 3 do not match the usual hydrogenosomal morphology, casting doubt on their identification as hydrogenosomes (as I noted previously). To resolve this, the authors should either use at least two antibodies for co-localization within the organelle or provide electron microscopy support, per my prior suggestion. Notably, no reference is provided for the source of the anti-Hmp24 antibody. Nevertheless, I appreciate the authors' clarification that Figure 3 primarily focuses on the cytoskeleton/tubulin. A straightforward solution would be to remove the Hmp24 signal from Figure 3 (along with related comments) and address hydrogenosome morphology in cysts in a future study.

-Type of response: Clarification and figure modification.

First, we would like to apologize to the reviewer for not having clearly addressed this point in our previous response. We appreciate the opportunity to clarify this issue. Although we performed immunofluorescence assays using both anti-Hmp24 and anti-Hmp35 antibodies (both kindly provided by Dr. Patricia Johnson, Department of Microbiology, Immunology & Molecular Genetics, University of California, Los Angeles, USA) and observed a similar labeling pattern in the images (data not shown), we acknowledge that immunofluorescence alone is insufficient to allow an in-depth evaluation of these structures during the encystment process of *T. foetus*. A more comprehensive characterization of these structures during encystment will require additional experimental approaches and will be addressed in future studies. In order to avoid potential misinterpretation, we have removed the corresponding image and related comments from the revised manuscript.

On the other hand, we acknowledge that the origin of the anti-Hmp24 antibody was not described in the main text of the manuscript, which constitutes a non-minor omission. In this regard, we would like to clarify that the description of the antibody source was provided in the NR-Reporting Summary submitted with the manuscript. We apologize for any confusion this may have caused and appreciate the reviewer for bringing this point to our

attention. Although the corresponding figure has now been removed, we recognize that the omission of this information in the main text was inappropriate.
